# Potential Substitutes for Replacement of Lead in Perovskite Solar Cells: A Review

**DOI:** 10.1002/gch2.201900050

**Published:** 2019-07-22

**Authors:** Ravinder Kour, Sandeep Arya, Sonali Verma, Jyoti Gupta, Pankaj Bandhoria, Vishal Bharti, Ram Datt, Vinay Gupta

**Affiliations:** ^1^ Department of Physics Government Degree College for Women Kathua Jammu and Kashmir 184102 India; ^2^ Department of Physics University of Jammu Jammu and Kashmir Jammu 180006 India; ^3^ Department of Physics Government Gandhi Memorial Science College Jammu Jammu and Kashmir Jammu 180001 India; ^4^ Departamento de Ciência dos Materiais Faculdade de Ciências e Tecnologia FCT Universidade Nova de Lisboa 2829‐516 Campus de Caparica Portugal; ^5^ Advance Materials and Devices Division CSIR‐National Physical Laboratory Dr. K. S. Krishnan Marg New Delhi 110012 India; ^6^ Department of Mechanical and Materials Engineering Khalifa University of Science and Technology Masdar Institute Masdar City 54224 Abu Dhabi UAE

**Keywords:** lead‐free perovskites, photovoltaic parameters, stability

## Abstract

Lead halide perovskites have displayed the highest solar power conversion efficiencies of 23% but the toxicity issues of these materials need to be addressed. Lead‐free perovskites have emerged as viable candidates for potential use as light harvesters to ensure clean and green photovoltaic technology. The substitution of lead by Sn, Ge, Bi, Sb, Cu and other potential candidates have reported efficiencies of up to 9%, but there is still a dire need to enhance their efficiencies and stability within the air. A comprehensive review is given on potential substitutes for lead‐free perovskites and their characteristic features like energy bandgaps and optical absorption as well as photovoltaic parameters like open‐circuit voltage (*V*_OC_), fill factor, short‐circuit current density (*J *_SC_), and the device architecture for their efficient use. Lead‐free perovskites do possess a suitable bandgap but have low efficiency. The use of additives has a significant effect on their efficiency and stability. The incorporation of cations like diethylammonium, phenylethyl ammonium, phenylethyl ammonium iodide, etc., or mixed cations at different compositions at the A‐site is reported with engineered bandgaps having significant efficiency and stability. Recent work on the advancement of lead‐free perovskites is also reviewed.

## 1 Introduction

**Figure 1:**
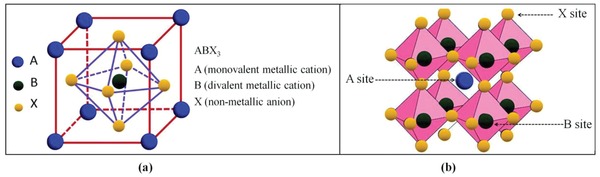
a) Schematic view of cubic perovskite crystal structure for ABX_3_ compound, b) 3D crystal structure in which the A site is confined within a cage determined by the octahedral coordination of B site with X site. Reproduced with permission.^1^ Copyright 2019, Royal Society of Chemistry.

Perovskite originally referred to a mineral calcium titanium oxide, CaTiO_3_, discovered in 1839 in Ural Mountains of Russia by Gustav Rose, a German mineralogist and later named after a Russian mineralogist count Lev Aleksevich Perovski.^1^ Since then, the term perovskite has been used for any organic/inorganic compound (synthetic/natural) with the similar crystal structure and stoichiometry as of CaTiO_3_, that is, ABX_3_, where A is monovalent metallic cation, most usually from group I of the periodic table. B is divalent metallic cation, a transition metal and X is a nonmetallic anion (halide). However, for O^2−^ anions, A and B are divalent and tetravalent cations, respectively. The size of cation A must be larger than that of cation B. Ideally, perovskite crystal structure is described as a body centered cubic structure with monovalent cation A dodecahedrally (12‐fold) coordinated by X anions as shown in **Figure** **1**.^2^ The volume occupied by A ions depends on the electronegativity and size of B and X ions, respectively. A superfluity of organic/inorganic compounds has been discovered that exists in perovskite crystal structure framework ABX_3_ like BaTiO_3_, SrTiO_3_, KNbO_3_, etc.

Generally, perovskite materials can be classified into two groups, namely, inorganic oxide perovskite and halide perovskite that further encompass alkali halide and organometal halide perovskite materials. There are some perovskite materials like MgCNi_3_ having neither oxygen nor halide component and hence do not belong to either of the groups.^3^ In alkali halide perovskites, A‐site is occupied by a monovalent organic cation such as CH_3_NH_3_ (methylammonium or MA), NH_2_(CH)NH_2_ (formamidinium or FA), or inorganic cations such as rubidium (Rb), caesium (Cs), etc., the B‐site by a divalent metal cation lead (Pb) or tin (Sn), and X‐site by a halide anion. In today's scientific world, it is the halide perovskites that have grabbed all the attention of silicon‐dominated photovoltaics industry and whole of the photovoltaics research is now focused in developing perovskite materials for solar energy conversion.

The suitability of a particular combination of cations to organize into a perovskite structure can be estimated based on two important parameters. The first one is the Goldschmidt tolerance factor (*t*), a dimensionless number, calculated from the ratio of ionic radii^4^

(1) $$t=\frac{r_{A}+r_{X}}{\sqrt{2}\left(r_{B}+r_{X}\right)}$$

where *r*_A_ and *r*_B_ are the ionic radii of cations A and B, and *r*_X_ is the ionic radius of anion. For a particular perovskite structure, the tolerance factor (*t*) can be calculated by substituting the ionic radii of cations and anions. If *t* = 1, it indicates the formation of an ideal cubic structure having size of cation A larger than that of B. The tolerance factor (*t*) must lie in the range of 0.8–1.0 for the formation of stable perovskite structures. If *t* < 0.8 or *t* > 1.0, the cation A is too small or too big to fit into BX_6_ octahedron, thereby resulting in the formation of alternative structures. The tolerance factor (*t*) leading to formation of different types of structures with examples is mentioned in **Table** **1**.

**Table 1:** Goldschmidt tolerance factor (*t*) of various perovskite materials^5^

Goldshmidt tolerance factor (*t*)	Structure	Example
0.9–1.0	Cubic	SrTiO_3_,^6^ BaTiO_3_ 7
0.71–0.9	Several perovskite structures/orthorhombic rhombohedral	GdFeO_3_, CaTiO_3_ 6
<0.71	Ilmenite	FeTiO_3_,^5^ KNbO_3_ 5
>1	Hexagonal or tetragonal	BaNiO_3_ 6

The second one is the octahedral factor (μ) which is the ratio between ionic radii of B and X

(2) $$\mu =\frac{r_{B}}{r_{X}}$$

The octahedral factor (μ) must lie in the range of 0.44–0.72 for B and X in order to form a stable BX_6_ octahedron.^2^ The tolerance factor has an immense role to play in finding alternative lead halide perovskite materials as many different cations can be inserted in ABX_3_ structure framework leading to development of varied materials with specific engineered properties.^8^

The effective ionic radii of organic molecular cations and Shannon ionic radii of inorganic cations as well as the effective ionic radii of various anions are listed in **Table** **2**.^9^, ^10^, ^11^, ^12^, ^13^

**Table 2:** (a) Effective ionic radii of organic molecular cations. (b) Shannon ionic radii of inorganic cations. (c) Effective ionic radii of various anions

(a)		
Cation A	Effective ionic radii (*r*_eff_) [pm]	Ref.
Ammonium [NH_4_]^+^	146	10
Hydrazinium [NH_3_NH_2_]^+^	217	10
Azetidinium [(CH)_3_NH_2_]^+^	250	10
Formamidinium [CH(NH_2_)_2_]^+^	253	10
Imidazolium [C_3_N_2_H_5_]^+^	258	10
Dimethylammonium [(CH)_2_NH_2_]^+^	272	10
Ethyl ammonium [(CH_3_CH_2_)NH_3_]^+^	274	10
Guanidinium [(NH_2_)_3_C]^+^	278	10
Tetramethylammonium [(CH_3_)_4_N]^+^	292	10
Thiazolium [C_3_H_4_NS]^+^	320	11
Tropylium [C_7_H_7_]^+^	333	11
Hydroxylamine [NH_3_OH]^+^	216	10
Methylammonium [CH_3_NH_3_]^+^	217	10
Piperazinium [C_4_H_12_N_2_]^2+^	322	9
Dabconium [C_4_H_14_N_2_]^2+^	339	9
K^+^	164	12
Rb^+^	172	12
Cs^+^	188	12
		
(b)		
Cation B	Effective ionic radii (*r*_eff_) [pm]	Ref.
Be^2+^	16	12
Mg^2+^	72	12
Ca^2+^	100	12
Sr^2+^	118	12
Ba^2+^	135	12
Mn^2+^	66	12
Fe^2+^	78	12
Co^2+^	58	12
Ni^2+^	55	12
Pd^2+^	86	12
Pt^2+^	60	12
Cu^2+^	73	12
Zn^2+^	60	12
Cd^2+^	78	12
Hg^2+^	69	12
Ge^2+^	73	12
Sn^2+^	110	13
Pb^2+^	119	12
Eu^2+^	117	12
Tm^2+^	103	12
Yb^2+^	103	12
Sn^[4+]^	69	12
Te^+^	150	12
Au^+^	137	12
Au^3+^	85	12
Sb^+^	76	12
Bi^3+^	103	12
Te^[4+]^	97	12
La^3+^	103	12
Ce^3+^	101	12
Pr^3+^	99	12
Nd^3+^	98	12
Sm^3+^	96	12
Eu^3+^	95	12
Gd^3+^	94	12
Dy^3+^	91	12
Er^3+^	89	12
Tm^3+^	88	12
Lu^3+^	86	12
Pu^3+^	100	12
Am^3+^	98	12
Bk^3+^	96	12
		
(c)		
Anion X	Effective ionic radii (*r*_eff_) [pm]	Ref.
Fluoride, F^−^	129	9
Chloride, Cl^[−]^	181	9
Bromide, Br^[−]^	196	9
Iodide, I^[−]^	220	10
Formate, HCOO^[−]^	136	9
BH_4_^[−]^	203	11

The Goldschmidt tolerance factor (*t*) has played a pivotal role in development of perovskites^10^ and is now being used to engineer/synthesize new organic–inorganic stable perovskites structures by formulating the composition of perovskite. The tolerance factor can be tuned to the stable perovskite range by mixing distinct A/B cations and X anions in a particular composition.^14^, ^15^, ^16^, ^17^

## 2 Perovskite Sensitized Solar Cell

Solar energy has always been sought to be converted into electrical energy through photovoltaic effect of light absorbing semiconductor in order to obtain clean and green energy. The traditional first generation crystalline silicon solar employed for this purpose enjoy a market share of more than 90% in PV market.^18^ The second generation solar cells consist of thin films such as cadmium telluride, copper indium gallium selenide, and amorphous silicon. The third generation has a number of thin film technologies such as dye‐sensitized solar cells (DSSCs) in development phase. The crystalline silicon solar cell has a theoretical limiting power efficiency of 33.16%^19^ noted as a Shockley Queisser limit in 1961. An efficiency of 25.6%^20^ for a silicon solar cell has been reported in 2014 that further grows to 46.1%^21^ in four‐junction GaInP/GaAs/GaInAsP/GaInAs solar cell reported by French‐German collaboration. The triple‐junction thin film solar cells achieved an efficiency of 13.6% in June 2015.^22^ The research teams at NREL, EPFL, and GSEM have reported Sun efficiencies of dual‐junction GaInP/GaAs solar cell devices up to 32.8%.^23^ Although, the monocrystalline silicon cells have photovoltaic conversion efficiency of more than 20%,^24^ they are characterized by high cost, difficult preparation conditions, and serious environmental pollution.^25^ Also, cadmium telluride and copper indium gallium selenium thin film solar cells' large‐scale use puts a pressure on environmental pollution. DSSCs showing an efficiency of more than 13% have low cost and easy fabrication but absorption layer in such cells is very thick^26^ and light dyes used in such cells suffer from phenomenon of light bleaching.

An efficient solar cell technology must ensure low raw material and finished material cost, high light absorption and solar power conversion efficiency, high abundance of raw material, low toxicity, and less environmental pollution. In order to achieve it, the organic/inorganic perovskite compounds can be used in light harvesting layer as these materials have all the requisite properties that make them suitable for use in PV^27^ applications. With the discovery of metal halide perovskite, especially MAPbI_3_, FAPbI_3_ as light absorbers, the use of perovskites in PV technology has been explored as they are cost effective and readily available for large‐scale use.^28^, ^29^, ^30^, ^31^, ^32^, ^33^, ^34^, ^35^, ^36^, ^37^, ^38^ The organic–inorganic perovskite materials have been pioneering in fabricating high solar power conversion efficiency hybrid solar cells from time to time.^32^, ^39^, ^40^, ^41^, ^42^, ^43^, ^44^, ^45^ Miyasaka and co‐workers reported the first perovskite sensitized solar cell (PSSC) in between 2006 and 2008 using CH_3_NH_3_PbI_3_ and CH_3_NH_3_PbBr_3_ absorbers and reported solar power conversion efficiency varying between 0.4 and 2% for solid‐state and liquid electrolyte cells, respectively.^46^, ^47^ A MAPbI_3_‐based solar cell with solar power conversion efficiency of 3.8% has been reported by Kojima et al. in 2009 and was the first peer reviewed publication on perovskite‐sensitized solar cell.^28^ Park and co‐workers using CH_3_NH_3_PbI_3_ liquid electrolyte solar cell reported an improved efficiency of 6.5%.^48^ In 2011, Snaith along with his co‐workers developed a solid‐state perovskite solar cell (PSC) using 2,2(7,7)‐tetrakis‐(N,N‐dimethoxyphenylamine)9,9(Spiro‐bifluorene) (Spiro‐OMeTAD) for hole transportation and produced solar power conversion efficiencies between 8 and 10%^49^ achieving a major breakthrough in performance efficiency of PSSC in comparison to DSSCs having only 7% efficiency.^50^ In 2012, Kim et al. replaced the liquid electrolyte with a solid‐state hole conducting material depositing the perovskite precursor over the mesoporous TiO_2_ layer achieving a solar power conversion efficiency (SPCE) of 9.7%.^30^ Later, increased efficiencies of 10.9% were reported by Lee et al.^51^ Gratzel and co‐workers reported a SPCE of 15% by using sequential deposition to produce pinhole‐free perovskite layer.^52^ Liu et al. Introduced Zn_2_SnO_4_ nanocrystalline thin film on PCBM buffer layer to make electron extraction process easy, thereby, increasing SPCE to 17.76.^53^ You et al. and Yang et al. first fabricated all metal oxide layer based perovskite solar cell reporting an efficiency of 16.1% and more stability of the material in 2016.^54^ Yang et al. and his team reported SPCE of 22.1%^55^ in defect engineered thin perovskite layers in PSSCs containing formamidinium with multiple cations and mixed halide anions in 2017. The SPCE of solid‐state PSSCs was around 10% in 2012 that later grew up to 22.1% in 2017. This has been achieved through engineering of perovskite composition and thin film deposition methods. The big issue of degradation of perovskite in polar liquid electrolyte has been solved by use of solid‐state PSSCs that have shown 500 h stability in ambient conditions without encapsulation but still the PSSCs have to prove its stability in air, on exposure to humidity,^56^, ^57^, ^58^ UV light,^59^ and high temperatures.^60^, ^61^ Research has also revealed that PSSCs also suffer from anomalous current–voltage hysteresis as reported by Snaith and co‐workers in 2014^62^ that can have adverse effects on the stability of PSSCs.^63^, ^64^

## 3 Device Configuration and Working Principles of Perovskite Solar Cell

**Figure 2:**
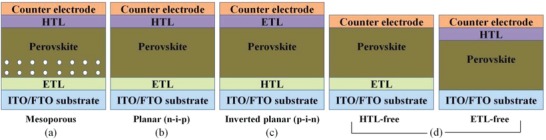
a) Device structure of mesoporous PSCs, b) planar heterojunction, c) inverted planar PCSs, and d) HTM‐free PSCs. Reproduced with permission.^66^ Copyright 2016, Springer Nature.

In the first perovskite solar cell fabricated in 2009, perovskite nanoparticles were used as a light absorber replacing dyes in dye‐sensitized solar cells. In the fabricated device, mesoporous TiO_2_ layer of several micrometer thickness acts as an anode and a platinum‐coated glass acted as a cathode in a liquid electrolyte based device.^28^, ^29^ However, the device suffered seriously from the stability issue as the perovskite light absorber layer dissolves or decomposes in the liquid electrolyte very rapidly. Hence, the liquid electrolyte was replaced by a solid‐state material to act as a hole transport material (HTM) resulting in a solid‐state mesoscopic perovskite solar cell with an improved stability. Organic Spiro‐OMeTAD was used as a hole transport material in such cells.^49^ The perovskite materials when used as a light absorber enhances the device stability and performance to its broad optical absorption range than the conventional dyes.^65^ In a mesoscopic perovskite solar cells, a compact metal oxide (TiO_2_) layer is deposited on a fluorine‐doped tin oxide (FTO) glass substrate by spin‐coating on which is further deposited a mesoporous TiO_2_ layer by spin‐coating. The perovskite light absorber layer is grown on the scaffold of mesoporous TiO_2_ layer which is further deposited by a HTM by spin‐coating and finally to a metal‐back electrode (Ag or Au). The device configuration of mesoporous perovskite solar cell is shown in **Figure** **2**a.^66^

TiO_2_ is most commonly employed in mesoporous layer that facilitates in the formation of inner connected layer of perovskite crystals by allowing their deep penetration into the pores of mesoporous layer. Compact TiO_2_ layer transports electrons, blocks holes, and suppresses the recombination of electron–hole pairs. The mesoporous TiO_2_ layer needs a high‐temperature sintering that can consequently increase the device fabrication time. Since the perovskite materials have ambipolar nature, they have the potential of transporting electrons and holes on their own in between two electrodes so a planar structure is viable for them.^67^ Also, the perovskite solar cells using planar structure over time have revealed the best device performance as that of a mesoporous structure.^32^, ^67^ The device with planar configuration has reported almost 100% internal quantum efficiencies ascertaining them as an efficient device structure.^43^ Thus, typically there are two major device configurations for a perovskite solar cell, viz., a planar heterojunction/conventional structure (n‐i‐p) and an inverted planar structure (p‐i‐n). In a planar heterojunction structure (n‐i‐p) as shown in Figure 2b,^66^ a compact electron transport layer (ETL) of 30–50 nm of TiO_2_ (most commonly) is deposited on a transparent conducting oxide substrate that can be indium‐doped tin oxide (ITO) or FTO. The mesoporous TiO_2_ layer is removed and perovskite light absorber to sandwich between an ETL and a hole transport layer (HTL) by spin‐coating or by vapor deposition and vapor‐assisted solution process on a compact TiO_2_ 32, 33 layer and finally connected to a metal electrode such as Au, Ag, or Pt. Spiro‐OMeTAD or poly‐triallylamine (PTAA) can be used in ETL. For an inverted planar structure (p‐i‐n) as shown in Figure 2c,^66^ a hole transport layer of poly(3,4‐ethylene dioxythiophene):poly(styrene sulfonate) (PEDOT:PSS) or NiO*_x_* is deposited on a conducting glass substrate that is most commonly ITO followed by a photoactive perovskite light absorber layer and is further covered by an electron transport layer of [6,6]‐phenyl‐C_61_‐butyric acid methyl ester (PC_61_BM) or zinc oxide (ZnO) and finally to a metal electrode of Au, Ag, or Al. The electron and holes are generated in the photoactive perovskite layer on absorption of photons of incident light and move to the opposite electrodes constituting current. HTL is used to receive the holes generated in the perovskite layer and transports them to the surface of the metal electrode whereas ETL transports electrons, block holes, and inhibits the electron–hole recombination in the FTO conductive substrate. The material used in ETL must be a n‐type semiconductor with high carrier mobilities, transparent to light, and with a suitable energy band structure matching with that of the perovskite material. ETL must have lowest unoccupied molecular orbital (LUMO) and highest occupied molecular orbital (HOMO) higher than the photoactive perovskite layer while HTL can facilitate hole motion only if the HOMO matches with the valence band of the perovskite material. The inverted planar structure has an operational edge over a conventional structure as it required a temperature of 300 °C for device fabrication in contrast to a planar heterojunction structure where a temperature up to 500 °C is required. Moreover, the hysteresis effect of perovskite solar cells is rarely observed in a planar inverted structure while this effect is most commonly observed in planar heterojunction devices.^68^ The highest device performance has been observed with the planar heterojunction structure using TiO_2_ as an electron transport layer.^69^ The most commonly reported structure is inverted planar device PEDOT:PSS/light absorber/PCBM as it is easily fabricated and more cost effective.^68^, ^70^ The poor SPCE of inverted planar structure may be due to a barrier at the contact interface between Fermi level of the metal electrode and lowest unoccupied molecular orbit of the ETL.^126^ In a planar heterojunction structure, the expensive HTL of Spiro‐OMeTAD may be removed leading to a new device framework known as planar HTM‐free architecture^71^, ^72^ as shown in Figure 2d.^66^

## 4 Why Lead‐Free?

The use of organic–inorganic lead halide perovskite such as MAPbI_3_ and FAPbI_3_ has caused an increase in solar power conversion efficiencies from 3.8% in 2009^28^ to 22.1%^34^, ^35^, ^73^, ^74^, ^75^, ^76^ in last nine years as these materials do possess requisite optoelectronic features such as a direct bandgap, long charge carrier lifetime, diffusion length, high charge carrier mobility, and strong optical absorption coefficient.^77^, ^78^, ^79^, ^80^, ^81^, ^82^, ^83^, ^84^, ^85^, ^86^, ^87^, ^88^, ^89^, ^90^ Lead halide perovskite has high open‐circuit voltages due to photon recycling as a result of which they have long charge extraction lengths through multiple absorption–emission events within the perovskite active layer.^91^ The metal lead has invaluable intrinsic properties like high melting point, high density, malleability, ductility, corrosion resistance, etc. Despite having all characteristic features to be exploited in commercial PV solar market, it is the toxic nature of lead in lead halide perovskite solar cells that hinders its use in silicon‐dominated PV market. The stringent directives of European Union clearly prohibits the use of hazardous substance in electrical and electronic equipment and lead has been identified as one of the ten hazardous chemicals listed by ROHS in order to avoid its exposure to environment and people as well.^92^, ^93^ The toxicity of lead is due to its affinity for band formation with thiol and cellular phosphate groups of numerous enzymes, proteins, and cell membranes.^94^ Lead halide perovskite solar cells do contain a considerable portion of lead, that is, 33% by weight. Lead is carcinogenic in nature and has no safe threshold limit of exposure. It can cause serious toxicological implications on human beings leading to cardiovascular and development diseases by inflicting the functioning of liver, kidney, brain, and central nervous system. Exposure to lead can produce irreversible health damages in infants and pregnant ladies.^95^, ^96^, ^97^ Also, organic–inorganic lead halide perovskites are liable to degradation under moisture, rain, heat, and prolonged illumination in air.^98^, ^99^, ^100^ Therefore, instability is another prime issue linked with these materials that reduces their working life span which is the most important prerequisite for commercialization on large scale as PV panels are generally placed over roof tops or in open fields so their exposure to rain is inevitable.

Hailegnaw et al. have reported that in case of a catastrophic failure of a solar plant, the impact of rain of different pHs on MAPbI_3_ films is complete degradation of perovskite material leaving behind PbI_2_ in water in the order of 10^−8^ mol L^−1^ which is of course low but higher than that of CdTe, CdS, and PbS values varying from 10^−27^ to 10^−34^ so it becomes most probable that lead, being soluble in water, may leech into the underground water resources.^101^ Not only this, Hailegnaw et al. have analyzed the impact of leaching lead out of the damaged solar panels on the soil and reported that the leakage of lead due to broken encapsulation or sealing will induce the concentration of Pb in first cm of ground below the damaged solar panel by 70 ppm.^101^ Taking into consideration the repercussions of use of toxic lead halide perovskites, it becomes pertinent to investigate lead‐free perovskite materials providing better stabilities with solar power conversion efficiencies without compromising human health and environment.

## 5 Characteristic Features of Lead‐Free Perovskites

The perovskite based materials used in solar cells do possess such a structure that enables them to have most suitable optical bandgaps to act as a light absorber. These materials do possess a high dielectric constant, long diffusion length, and a broad optical absorption range covering the entire visible spectrum and into the infrared. Perovskite materials exhibit ambipolar properties that enable them to display both n‐type and p‐type behavior on exposure to photons of incident light. The rate of nonradiative recombination in such material is strongly suppressed that is essential for high solar power conversion efficiencies. The presence of hysteresis loss in these materials clearly indicates the presence of magnetic properties at room temperature or above. Another important characteristic of these materials is that they are deposited by low‐temperature solution methods that provide easy fabrication with low production cost. Besides they are typically flexible, light weight, and semitransparent making them more appealing for use in photovoltaic applications.

The lead‐based halide perovskites have reported a highest solar power conversion efficiency of 22% up to now within 8 years of research.^102^ The efficiency limit of perovskite solar cell has been envisaged to be 31% based on detailed balance calculations much closer to the Shockley–Queisser limit of 33%.^103^ Although the lead‐based halide perovskites have all the structural, optical, and electrical features for use in perovskite solar cell as a light absorber but due to toxicity issues of lead, it is pertinent to replace it by another suitable elements such as tin, germanium, bismuth, etc. The lead‐free perovskites have attracted the attention of the researchers at present time due to significant properties of these materials that can be engineered to make them suitable for their use as light absorbers in perovskite solar cells. Lead‐free perovskite methylammonium tin iodide MASnI_3_ is a direct gap semiconductor with an optical bandgap of 1.3 eV^104^, ^105^, ^106^ which is close to 1.5 eV of MAPbI_3_. It exhibits a strong photoluminescence emission corresponding to the onset at 950 nm in 700–1000 nm range of the absorption edge at room temperature. MASnI_3_ has an electrical conductivity of 5 × 10^−2^ S cm^−1^ at room temperature that corresponds to a Seebeck coefficient of ≈–60 µV K^−1^. The material exhibits a carrier concentration of the order of ≈1 × 10^14^ cm^−3^ having excellent electron mobilities of the order of ≈2000 cm^2^ V^−1^ s^−1^ and hole mobility of 300 cm^2^ V^−1^ s^−1^ in comparison to lead halide perovskites that have an electron mobility of 66 cm^2^ V^−1^ s^−1^ and hole mobility of 105 cm^2^ V^−1^ s^−1^.^84^ **Table** **3** summarizes the electron and hole mobilities of all the lead‐free perovskite materials. The Hall measurements of as‐grown crystals of MASnI_3_ have revealed a hole concentration of about 9 × 10^17^cm^−3^ with a hole mobility of about 200 cm^2^ V^−1^ s^−1^ at 250 K.^107^

**Table 3:** Charge carrier mobility of lead‐free perovskites

Light absorber	Architecture	Measurement method	Component	Mobilities [cm^−1^ V^−1^ s^−1^]	Ref.
MASnI_3_	Poly c	Hall	Electron	2320	105
MASnI_3_	Poly c	Hall	Hole	322	105
MASnI_3_	Single c	Hall	Hole	200	107
MASnI_3_	Poly c	Hall	Hole	50	121
MASnI_3_	Mesostructural	THzC	Total	1.6	120
FASnI_3_	Poly c	Hall	Electron	103	105
FASnI_3_	Film	THzC	Total	22	122
CsSnI_3_	Poly c	Hall	Electron	536	105
CsSnI_3_	Poly c	Hall	Hole	585	123
(C_6_H_5_C_2_H_4_NH_3_)_2_SnI_4_	Film	FET	Hole	0.6	124
PEASnI_4_	Film	FET	Hole	15	125
(MA)_3_Sb_2_I*_x_*Br_9−_ *_x_*	Single c	SCLC	Electron	12.3	116
(MA)_3_Sb_2_I*_x_*Br_9−_ *_x_*	Single c	SCLC	Hole	4.8	116
Ba_2_BiTaO_6_	Film	Hall	Hole	30	126
MASnI_3_	Mesostructural	–	Electron	2000	106
MBI	Single c	Hall	Total	1–11	127
(MA)_3_Sb_2_I_9_	Single c	SCLC	Hole	4.8	116
(MA)_3_Sb_2_I_9_	Single c	SCLC	Electron	12.3	116
(MA)_3_Sb_2_I_9_	Film	SCLC	Hole	1.2 × 10^−4^	116
(MA)_3_Sb_2_I_9_	Film	SCLC	Electron	1.5 × 10^−4^	116
Cs_2_SnI_6_	Poly c	Hall	Hole	310	115
MA_3_Bi_2_I_9_	Single c	SCLC	Hole	29.7	128
MA_3_Bi_2_I_9_	Single c	Hall	Electron	1	127
(MA)_2_SnI_6_	Poly c	Hall	Electron	3	129

Although tin halide perovskite has higher charge carrier mobilities, Sn^2+^ has a strong tendency to get oxidized to Sn^4+^ causing a p‐type self‐doping.^108^ The artificial hole doping of the halide‐based perovskites increases their electrical conductivity and they exhibit a metal‐like conducting behavior.^107^ The formamidinium tin iodide (FASnI_3_) has an optical bandgap of 1.41 eV that is much closer to the bandgap 1.5 eV of MAPbI_3_ making it a potential candidate to display an optical absorption up to 950 nm.^109^ The cesium tin iodides (CsSnI_3_) display a bandgap of ≈1.3 eV at 300 K close to the optimum value of 1.5 eV for photovoltaic performance.^110^ Bismuth‐based halide perovskites display lower light absorption onset at 450 nm with absorption coefficient of ≈1 × 10^5^ cm^−1^ that are lower as compared to MAPbI_3_ that has an absorption coefficient of around 2 × 10^5^ cm^−1^ at 450 nm.^111^

Lead‐free perovskites have high exciton binding energies that provide them stable optical properties. The exciton binding energies of bismuth‐based halide perovskites MA_3_Bi_2_I_9_, Cs_3_Bi_2_I_9_, and MA_3_Bi_2_I_9_Cl*_x_* are of 70, 270, and 300 meV that are much higher than that of lead‐based halide perovskites (25–50 meV).^111^ UV–vis absorption measurements for Cs_3_Bi_2_I_9_ have reported a strong exciton absorption peak at room temperature. Cs_3_Bi_2_I_9_ exhibits an exciton absorption peak at ≈485 nm (2.56 eV) with an indirect optical bandgap of ≈2.1 eV. The films exhibited an optical absorption coefficient of ≈1 × 10^4^ cm^−1^ at 450 nm. In spite of indirect bandgaps, the material is still a potential candidate for use as a light absorber due to strong exciton binding energy.^112^ CsSnI_3_ perovskite exhibits a direct bandgap of 1.32 eV with an exciton binding energy of 18 meV at room temperature. The large binding energy is on the account of exciton motion in the 2D layer of SnI_4_ tetragons present in the material.^123^

Tin‐based perovskites are prepared by using solution methods and crystallizes at room temperature whereas lead‐based halide perovskites crystallize by heating. The variation in composition of halide anion in lead‐free perovskites has a significant effect on the absorption coefficient of these materials thus paving the way for engineering the bandgaps and optical absorption spectrum of these materials. The tin‐based hybrid halide perovskites MASnI_3−_ *_x_*Br*_x_* (*x* = 0, 1, 2, 3) synthesized in an inert atmosphere in the nitrogen glove box exhibit an optical absorption onset that can be blueshifted from 954 to 577 nm by varying the composition of halide anion, that is, for *x* = 0 and *x* = 3 whereas for *x* = 1 and 2, optical absorption onset at 795 and 708 nm has been reported. Also ultraviolet photoelectron spectroscopy (UPS) measurements of valence band energy *E*_VB_ of MASnI_3−_ *_x_*Br*_x_* under high vacuum have revealed that the bandgaps can be engineered from 1.30 eV for MASnI_3_ to 2.15 eV for MASnBr_3_.^106^ Not only this, the color of the tin‐based hybrid halide perovskite MASnI_3−_ *_x_*Br*_x_* shows a variation with increased bromine content from black (*x* = 0) to dark brown (*x* = 1) and yellow (*x* = 3); thus, colorful solar devices can be designed by using bandgap engineering. Thus, the composition of tin‐based mixed halide perovskite can be tailored to emit between 954 and 574 nm in contrast to lead‐based counterparts that display photovoltaic emission in between 700 and 800 nm. The emitted wavelengths are in agreement with the values of bandgaps obtained through experiments clearly indicating the presence of direct optical bandgaps in MASnI_3_.^106^

The investigation of Ge mixed halide perovskites MAGeI_3−_ *_x_*Cl*_x_* [*x* = 0, 1, 1.5, 2, 3] by using first principle calculations has reported a bandgap of 1.8 eV for MAGeI_3_ (*x* = 0) whereas MAGeCl_3_ (*x* = 3) has a much wider bandgap of 3.8 eV clearly demonstrating the effect of doped chlorine in MAGeI_3_ perovskites. The absorption coefficients also display an increasing trend when the proportion of *x* decreases from 3 to 0 attributed to the redshift of the optical bandgap caused due to change in chemical composition of the material.^113^ In case of antimony‐based mixed halide perovskites MA_3_Sb_2_I*_x_*Br_9−_ *_x_*, the optical bandgap onset for perovskite films shows a decreasing trend, that is, the optical absorption onset is blueshifted from 558 to 453 nm as *x* changes from 9 to 0. The hole and electron mobilities of MA_3_Sb_2_I_9_ single crystals have been calculated by using space charge limited current methods^114^, ^115^ and are shown in Table 3. The MA_3_Sb_2_I_9_ single crystals have high absorption coefficient greater than 10^5^ cm^−1^ at absorption peak wavelengths. The absorption onset for [*x* = 0, 3, 6] in MA_3_Sb_2_I*_x_*Br_9−_ *_x_* films are 453, 486, and 516 nm with a direct bandgap of 2.78, 2.66, and 2.49 eV, respectively.^116^ Lead‐free perovskites do possess suitable carrier diffusion lengths and minority charge carrier lifetimes exhibiting photovoltaic performance. The long carrier diffusion lengths of electrons and holes in MASnI_3_ are 279 ± 88 and 193 ± 46 nm, respectively, obtained by broadband transient absorption and time‐resolved fluorescence spectroscopy. Addition of SnF_2_ in MASnI_3_ films results in not only increase in diffusion lengths to more than 500 µm but also enhances the fluorescence lifetime up to ten times.^117^ The background concentration of doped holes has an effect on the diffusion lengths of MASnI_3_ perovskite. As the background doping level in MASnI_3_ decreases, there is a corresponding increase in diffusion length. For a doping concentration below 10^15^ cm^−3^, the diffusion length can be engineered to increase above 1 µm in length that is close to the value shown by lead‐based halides.^120^ Lead‐free CsSnI_3_ perovskite films synthesized by the solution method have carrier lifetime of ≈54 ps, minority carrier diffusion length of ≈1.6 nm, and a doping concentration of more than 9.2 × 10^18^ cm^−3^ obtained as a consequence of better quality of crystalline films whereas single crystals of CsSnI_3_ have a long minority carrier diffusion length of more than 930 nm which is comparable to that of the lead‐based perovskites^110^ having diffusion lengths exceeding 1 µm.^74^

## 6 Hole Transport Material and Electron Transport Material in Lead‐Free Perovskite Solar Cells

**Figure 3:**
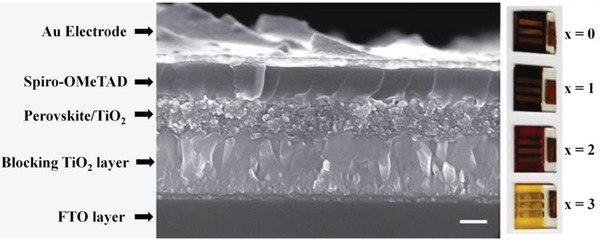
SEM image of a photovoltaic device using CH_3_NH_3_SnI_3_ perovskite material. Reproduced with permission.^106^ Copyright 2014, Springer Nature.

Lead‐free perovskites have been prepared by using mesoporous perovskite solar cells in planar heterojunction and inverted planar structures. Spiro‐OMeTAD is most commonly used hole transport material in lead‐free perovskites as it has the ability to penetrate deep into the pores of the perovskite layer but it has a low hole mobility and complicated device processing. Also it deteriorates the stability of the fabricated device.^130^, ^131^ Therefore, dopants are added into it in order to enhance its conductivity. The first lead‐free perovskite device was prepared by solvent engineering method by employing MASnI_3_ as a light absorber. Spiro‐OMeTAD has been used as a HTM on the top of the perovskite layer in a device architecture of FTO/compact TiO_2_/mesoporous TiO_2_ layer/MASnI_3_ light absorber/Spiro‐OMeTAD/Au.^120^ An additive doping of hydrogen bis(trifluoromethane sulfonyl)imide (H‐TFSI) and *tert*‐butyl pyridine is done into Spiro‐OMeTAD to enhance the rate of hole extraction and transport.^132^ The additive doping of lithium bis(trifluoro methyl sulfonyl)imide salt (Li‐TFSI) and 4‐*tert*‐butyl pyridine (TBP) deteriorates the stability of MASnI_3_ perovskite device than H‐TFSI.^120^ In another approach, also Spiro‐OMeTAD used as a HTM is doped with lithium bis(trifluoro methyl sulfonyl)imide and 2,6‐lutidine in order to enhance its hole mobility.^49^ The solar cell capacitance simulator and analytical calculations (SCAPS) have reported an efficiency of above 15% in lead‐free tin‐based MASnI_3_ perovskites employing Spiro‐OMeTAD as a hole transport material.^133^ Chlorobenzene (CB), Li‐TFSI, and TBP have been used as an additive in Spiro‐OMeTAD in lead‐free Ma_3_Bi_2_I_9_ perovskites.^111^ Oxygen‐doped Spiro‐OMeTAD employed as a HTM in Cs_3_Bi_2_I_9_ perovskite solar device has yielded the maximum of the reported solar power conversion efficiencies.^134^ The Spiro‐OMeTAD as a HTM has been used in lead‐free tin, germanium, antimony, bismuth, and copper‐based perovskite devices. **Figure** **3** shows the scanning electron microscopy (SEM) image of a MASnI_3_ perovskite device with Spiro‐OMeTAD as a HTM.^106^

Cu‐based lead‐free perovskites reported so far have a planar heterojunction (n‐i‐p) structure employing Spiro‐OMeTAD as a HTM with a highest reported efficiency of 2.41%. The low efficiency is attributed to the mismatch in the energy levels between the (MA)_2_CuCl*_x_*Br_4−_ *_x_* and Spiro‐OMeTAD as a HTM leading to a poor hole extraction in the device.^135^, ^136^, ^137^ Another polymeric organic HTM PTAA has been employed in planar heterojunction n‐i‐p perovskite devices.^138^, ^139^ Owing to its large hole mobility the use of PTAA as a HTM in Cs_3_Sb_2_I_9_ perovskite solar cells has reported a *V*_OC_ of 250–300 meV and an extremely low solar power conversion efficiency.^140^ The doping of bismuth‐based perovskite Cs_3_Bi_2_I_9_ films with N,N‐dimethyl formamide/hydroiodide (HI) solution featured a pure crystalline film with an excellent thermal stability.^141^ PTAA employed as a HTM in ethylene diammonium and methylammonium tin iodide en[MASnI_3_] has reported a SPCE of 6.63% with a very high current density of 24.3 mA cm^−2^ in a device architecture of FTO/C‐TiO_2_/mp‐TiO_2_/en[MASnI_3_]/PTAA/Au.^142^ Many research groups have synthesized lead‐free MASnI_3_, FASnI_3_, and CsSnI_3_ perovskite solar devices by using PTAA as a HTM. The presence of another polymeric organic poly(3‐hexyl thiophene) (P3HT) as a HTM in lead‐free perovskites can enhance the SPCE as well as stability of the fabricated device due to its potential to decrease the resistance from the hole transfer impedance. P3HT has been employed as a HTM instead of Spiro‐OMeTAD for Cs_3_Bi_2_I_9_ perovskite solar devices.^111^ In another research, P3HT is used as a HTM in thin films of CsBi_3_I_10_ perovskite deposited by solution processing in device architecture of glass/FTO/compact TiO_2_/mesoporous TiO_2_/CsBi_3_I_10_ light absorber/P3HT/Ag. The addition of dopant 4‐*tert*‐butyl pyridine in Spiro‐OMeTAD employed as a HTM in CsBi_3_I_10_ perovskite dissolves the light absorbing perovskite layer^143^ and suffers from stability and degradation issues. Unlike Spiro‐OMeTAD, P3HT enhances the SPCE of the fabricated perovskite device as in CsSnBr_3_ films, an efficiency of 0.11% was enhanced to 3.2% by replacing Spiro‐OMeTAD HTM by P3HT.^144^ Also, P3HT employed as a HTM in MA_3_Bi_2_I_9_ films enhance the overall performance of the fabricated device.^127^ P3HT has been employed as a HTM in MASnBr_3_, FASnI_3_, CsSnI_3_, en[MASnI_3_], Cs_2_SnI_6_, MA_3_Bi_2_I_9_, Cs_3_Bi_2_I_9_, CsBi_3_I_10_, AgBi_2_I_7_, and AgBiI_5_ perovskite solar devices.

**Figure 4:**
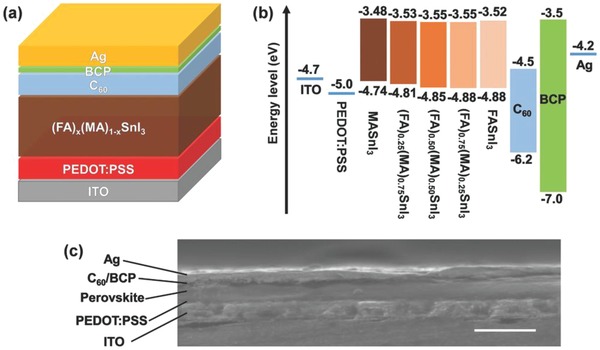
a) Schematic device structure of (FA)*_x_*(MA)_1−_ *_x_*SnI_3_ perovskite solar cell. b) Band alignment diagram. c) Cross‐sectional scanning electron microscope (SEM) image of a completed device (scale bar: 500 nm). Reproduced with permission.^150^ Copyright 2017, Wiley‐VCH.

In an inverted planar (p‐i‐n) perovskite device, the hole transport layer is kept under the perovskite light absorber layer that alleviates the stringent requirement of efficient conductivity of hole transport material. Polymeric organic HTM PEDOT:PSS is used in such devices. PEDOT:PSS is used as a HTM in FASnI_3_ perovskite solar cells in an inverted planar (p‐i‐n) architecture and at present has reported a maximum SPCE of 8.12% in FA_0.75_MA_0.25_SnI_3_ in lead‐free tin‐based perovskite with a *V*_OC_ of 0.61 V.^145^ The PEDOT:PSS with intercalated polyethylene glycol (PEG) used as a HTM in FASnI_3_ perovskite solar cell alleviates the energy level mismatch between the perovskite light absorber and PEDOT:PSS as HTM. As a consequence, the SPCE increased from 2.01 to 5.12% in the forward scan.^146^ The inverted planar (p‐i‐n) device structure employed for antimony‐based MA_3_Sb_2_I_9_ perovskite films has reported a SPCE of 0.5% with a *V*_OC_ of 0.89 V.^147^ Additives are added into PEDOT:PSS in order to enhance the conductivity and morphology of PEDOT:PSS films. Polyorganic solvents such as dimethyl sulfoxide (DMSO), dimethylformamide (DMF), and ethylene glycol have been used as additives in lead‐free perovskite device fabrication.^148^ The addition of additive in PEDOT:PSS films leads to enhancement in efficient hole extraction and collection rate attributed to the strong dipole–dipole or dipole–charge interactions between the polar additive and PEDOT:PSS used as a HTM in fabricated perovskite device.^149^ PEDOT:PSS as a HTM has been employed in lead‐free MASnI_3_, FASnI_3_, FASnI_3_Br, FA_1−_ *_x_*MA*_x_*SnI_3_, MA_3_Bi_2_I_9_, MA_3_Sb_2_I_9_, and Cs_3_Sb_2_I_9_ reported perovskite solar devices. **Figure** **4** shows the device structure and energy band diagram of (FA)*_x_*(MA)_1−_ *_x_*SnI_3_ perovskite solar cell.^150^ The use of Spiro‐OMeTAD as a HTM damages the perovskite film. In order to overcome this issue, inverted planar (p‐i‐n) perovskite solar cells of MASnI_3_ have been prepared by using PEDOT:PSS doped with poly‐TPD as a HTM as shown in Figure 4.^150^ Figure 4 shows the (a) schematic device structure of (FA)*_x_*(MA)_1−_ *_x_*SnI_3_ perovskite solar cell, (b) band alignment diagram, and (c) cross‐sectional SEM image of a completed device (scale bar: 500 nm).^[150]^ Addition of poly‐TPD layer into PEDOT:PSS resulted into suppressed charge recombination and better efficiencies.^151^

**Figure 5:**
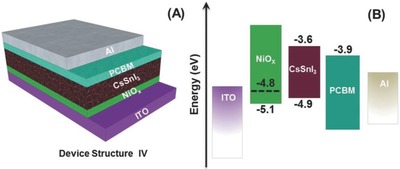
A) Scheme of the “inverted” structure planar B‐γ‐CsSnI_3_ PSC device employing NiO*_x_* as HTL and PCBM as ETL, and B) corresponding energy level diagram (the dashed line indicates NiO*_x_* work function). Reproduced with permission.^157^ Copyright 2016, Wiley‐VCH.

Besides organic HTMs, inorganic NiO (nickel oxide) and CuI (copper iodide) have also been used as a HTM in inverted planar (p‐i‐n) structures. NiO has a large work function of 15.2 eV in comparison to 5.2 eV for PEDOT:PSS that makes it viable for use as a HTM in perovskite solar cells reporting a higher *V*_OC_.^152^, ^153^, ^154^, ^155^ The thickness and morphology of NiO as a HTM has a direct impact on charge collection and recombination in perovskite solar devices. The use of NiO as a HTM leads to more air stability in FASnI_3_ perovskite inverted planar (p‐i‐n) solar cell using (PEA)_2_FA_8_Sn_9_I_28_ as a light absorber reporting a SPCE of 5.94%.^156^ NiO*_x_* has been employed as a HTM instead of Spiro‐OMeTAD in inverted planar structured B‐ϒ‐CsSnI_3_ PSCs in order to overcome the low conductivity of the undoped Spiro‐OMeTAD. The perovskite device exhibited an enhanced SPCE of 2.61% higher than that of Spiro‐OMeTAD used as a HTM.^157^ **Figure** **5** shows the device structure and corresponding energy diagram employing NiO*_x_* as a HTM in B‐ϒ‐CsSnI_3_ perovskite solar cells.^157^

**Figure 6:**
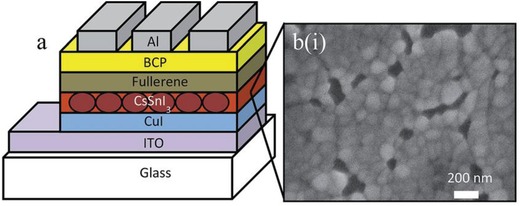
a) Schematic of the device architecture used in this work; b) SEM image of a CsSnI_3_ film prepared with a 10 mol% excess SnI_2_ and spin cast at 4000 rpm from 8 wt% solution onto an ITO glass substrate coated with a 100 nm layer of CuI. Reproduced with permission.^159^ Copyright 2015, Royal Society of Chemistry.

CuI has a hole conductivity greater than that of Spiro‐OMeTAD that enables CuI to improve the fill factor (FF) of the perovskite device employing it as a HTM.^158^ CuI has been used as a HTM in fabrication of CsSnI_3_ perovskite solar cells reporting a *V*_OC_ of 0.55 V and enhanced air stability.^159^ **Figure** **6** shows the device architecture of CsSnI_3_ using CuI as a HTM and SEM image of CsSnI_3_ films.^159^

**Figure 7:**
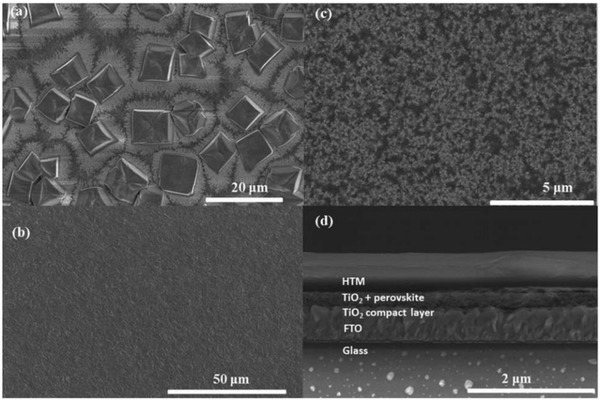
SEM images. a) Top view of a film of CH_3_NH_3_SnI_3_ spin‐coated onto mesoporous TiO_2_ (80 nm thickness). b) Top view of a spin‐coated film of CH_3_NH_3_PbI_3−_ *_x_*Cl*_x_* on mesoporous TiO_2_ (400 nm thickness). c) Top view of a spin‐coated film of CH_3_NH_3_SnI_3_ on mesoporous TiO_2_ (400 nm thickness). d) Cross‐sectional view of a complete device active layer composed of FTO glass/compact TiO_2_ (50 nm)/mesoporous TiO_2_ infiltrated with CH_3_NH_3_SnI_3_ (400 nm)/Spiro‐OMeTAD (600 nm). Reproduced with permission.^120^ Copyright 2014, Royal Society of Chemistry.

**Figure 8:**
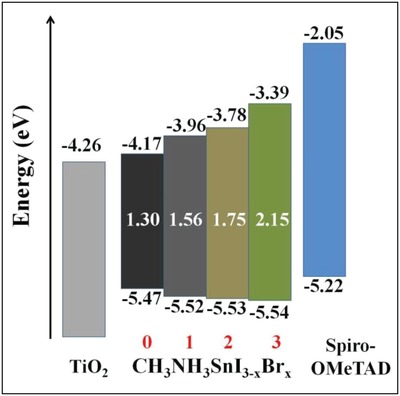
Schematic energy‐level diagram of CH_3_NH_3_SnI_3−_ *_x_*Br*_x_* compounds. Reproduced with permission.^106^ Copyright 2014, Springer Nature.

Perovskite solar cells without a HTM layer have the advantage of having simple structures, easy fabrication process, and higher stability if the work function of the metal electrode used in perovskite solar cells is close to the maximum valence band of perovskite light absorber, then absence of hole transport layer has no impact on the built‐in electric field.^160^ The perovskite material in HTL‐free devices works as a light absorber and a hole transport layer in such cells.^161^ A HTL‐free solar cell of MASnI_3_ has reported an efficiency of 3.15%, *J*_SC_ of 21.4 mA cm^−2^ that has been prepared through a solvent engineering method having a device architecture of the form FTO/c‐TiO_2_/mp‐TiO_2_/MASnI_3_ light absorber/Au.^162^ HTL‐free CsSnI_3_ PSC has stability ten times greater than the devices using same device architecture using MAPbI_3_ as a light absorber.^163^ Inorganic metal oxides like TiO_2_, ZnO, SnO_2_ and organic fullerene derivatives like phenyl‐C_60_‐butynic and methyl ester (PC_60_BM) or PC_70_BM have been employed as an ETM for perovskite solar cells. The efficient ETM should have the capability to engineer the optical bandgap for maximum absorption of incident light by the perovskite light absorber layer and must have a better electron extraction and hole blocking property in order to suppress the electron–hole recombination at the interface of the device. TiO_2_ as an ETM has been employed in device fabrication of most of the reported lead‐free perovskite solar cells. On the top of a mesoporous TiO_2_ layer, the perovskite film of MASnI_3_ is crystallized upon spin‐coating and it penetrates into the pores of ETM. The MASnI_3_ films fabricated on the top of 400 nm thick mp‐TiO_2_ layer are better than that of prepared on an 80 nm thick mp‐TiO_2_ layer. The mesoporous MAPbI_3−_ *_x_*Cl*_x_* perovskite films have a better film morphology than that of MASnI_3_ films fabricated in a similar way and architecture as shown in **Figure** **7**.^120^ **Figure** **8** shows the schematic energy‐level diagram of CH_3_NH_3_SnI_3−_ *_x_*Br*_x_* compounds.^106^

By controlled crystallization, it is possible to enhance the quality of film formation.^120^ In another approach, solvent engineering method was employed to prepare thin films of MASnI_3_. A 30 nm thick TiO_2_ compact layer as an ETM is deposited on the substrate by atomic layer deposition system. The perovskite light absorber crystals infiltrate into the pores of mp‐TiO_2_ layer and remaining pores of mesoporous TiO_2_ layer are filled up by the HTM forming a 200 nm thick capping layer on the top of the composite structure.^106^

In a planar heterojunction (n‐i‐p) structure, a compact TiO_2_ layer is deposited on a glass that is further covered by a mesoporous TiO_2_ layer in order to enhance the electron collection and to avoid hysteresis loss during *V*–*I* measurements.^164^ By employing mp‐TiO_2_ as an ETM, the homogenous MASnI_3_ films prepared by vapor‐assisted solution process^165^, ^166^ have reported a *J*_SC_ of 17.4 mA cm^−2^ when used as a light absorber in perovskite solar cells. The SPCE of pristine FASnI_3_ films was 0.003% by using mesoporous TiO_2_ layer as an ETM. The low value of SPCE is attributed to high background carrier density of 10^19^ cm^−3^ that leads to a metal like conductivity and device short circuiting.^156^ However, the addition of Br_2_ into FASnI_3_ films lowers the background carrier density of the perovskite. As a consequence of reduction in tin vacancies, the leakage current of the device is reduced that further increases the recombination lifetime and finally *V*_OC_ and FF of the fabricated device and SPCE up to 5.5%.^167^ TiO_2_ as an ETM has an intrinsic low mobility and this has been a generation of deep traps by UV light that results in charge accumulation, recombination classes, and severe *V–I* hysteresis.^41^, ^59^, ^62^, ^168^, ^169^ The evaporation‐assisted method combining thermal evaporation with solution method has been employed to obtain uniform, full coverage, dense, and pinhole‐free CsSnI_3_ films eliminating the direct contact between HTM and ETM and reduces the consequent recombination. Evaporation‐assisted solution method makes it feasible for convenient tuning of SnF_2_ addition as a solvent. The conventional mesoporous n‐i‐p structure PSCs with an architecture FTO/bl‐TiO_2_/mp‐TiO_2_/CsSnI_3_/OMeTAD/Au has reported an efficiency of 2.2% in the device based on a 66 nm thickness of CsI.^170^ In CsSnBr_3_, the best reported SPCE so far is 2.1% that is due to the significant role of SnF_2_ addition as a solvent that alleviates the serious mismatch of band energy levels between the perovskite light absorber and TiO_2_ layer as an ETM.^102^

**Figure 9:**
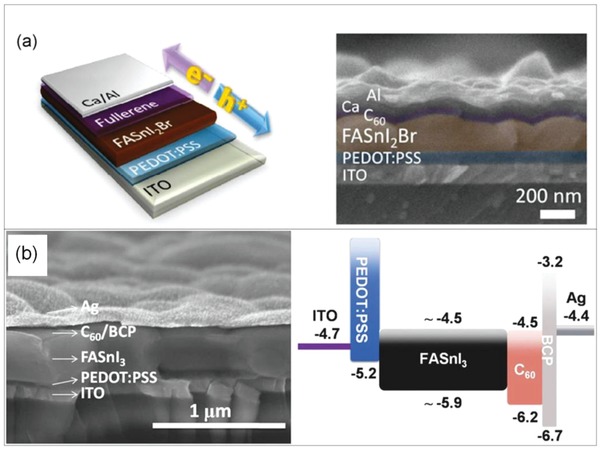
a) Configuration of the FASnI_2_Br‐based p‐i‐n heterojunction solar cells and its cross‐sectional SEM image of a typical device. Reproduced with permission.^171^ Copyright 2016, Springer Nature. b) Cross‐sectional SEM image of the entire device with 10 mol% SnF_2_ additives, in which each layer is labeled, and schematic of energy level diagram of our FASnI_3_ perovskite solar cells. Reproduced with permission.^172^ Copyright 2016, Wiley‐VCH.

Germanium‐based perovskites have been fabricated by employing compact and mesoporous TiO_2_ layer as ETM and Spiro‐OMeTAD as a HTM. The fabrication films of CsGeI_3_ and MAGeI_3_ displayed a smooth morphology with a SPCE of 0.11 and 0.20% whereas that of FAGeI_3_ exhibited a poor morphology leading to no photovoltaic behavior. The poor performance of the device is attributed to oxidation of Ge^2+^ into Ge^4+^ during fabrication process.^144^ However, TiO_2_ requires a high‐temperature sintering and exhibits degradation in SPCE on exposure to UV light. TiO_2_ requires high‐temperature annealing but the substrate cannot withstand such a high temperature. The mesoporous TiO_2_ (n‐i‐p) devices have exhibited better efficiencies whereas inverted planar (p‐i‐n) devices suffer from hysteresis losses. Tin‐based lead‐free perovskites are considered unsuitable for planar heterojunction solar cells due to their short diffusion lengths, a SPCE of 1.72% is shown by FASnI_2_Br films as a light absorber with C_60_ as ETM suggesting the significance of perovskite film morphology on the device performance. FASnI_2_Br films with an architecture ITO/PEDOT:PSS/FASnI_2_Br/ C_60_/Ca/Al reported a *J*_SC_ of 6.82 mA cm^−2^ and *V*_OC_ of 0.46 mV.^171^ **Figure** **9** shows the structure of (a) FASnI_2_Br (SEM image)^171^ and (b) FASnI_3_ (SEM image) and energy band diagram.^172^

**Figure 10:**
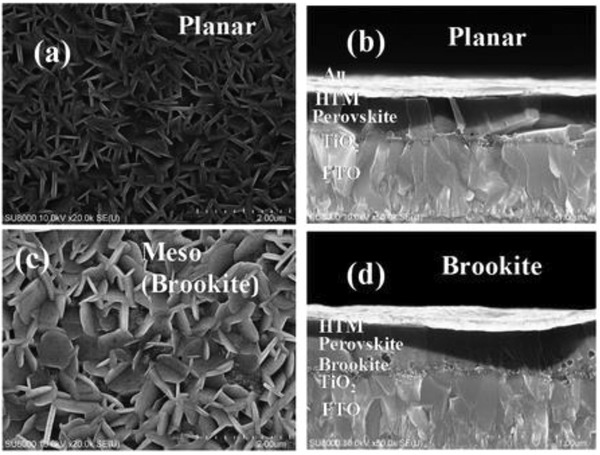
Top and cross‐sectional SEM view of MBI perovskite layer deposited on a,b) TiO_2_ compact layer and c,d) brookite mesoporous layer. Reproduced with permission.^174^ Copyright 2016, American Chemical Society.

Anatase, rutile, and brookite are three stable phases in TiO_2_ when used as an ETM. For anatase TiO_2_‐based perovskite solar cells, the electron diffusion constant was ten times higher but the time constant for recombination was ten times lower than for rutile TiO_2_‐based one. Fast charge recombination in anatase TiO_2_‐based device is the result of poor charge separation in TiO_2_/perovskite interface.^173^ **Figure** **10** shows the MBI perovskite layer deposited on a compact, mesoporous, and brookite TiO_2_.^174^ Fullerene C_60_ and its derivatives such as PC_60_BM or PC_70_BM have been employed as an interfacial material at the interface between TiO_2_ and perovskite layer because of its high electron mobility. A self‐assembled C_60_ monolayer was introduced on TiO_2_ surface that enhances the charge separation, reduces the capacitance of TiO_2_ and *V*–*I* hysteresis.^175^ Organic ETM of PC_60_BM or PC_70_BM is more efficient to collect electrons in comparison to mp‐TiO_2_ in inverted planar p‐i‐n devices as they can passivate the charge traps of metal oxide^176^, ^177^, ^178^ and hence can reduce the nonradiative recombination channels at the surface leading to an improved SPCE with a very low hysteresis.^179^, ^180^ The perovskite layer of Cs_3_Sb_2_I_9_ was prepared through a single‐step spin‐coating process for an inverted planar p‐i‐n structure using architecture glass/ITO/PEDOT:PSS/Cs_3_Sb_2_I_9_/PC_71_BM/C_60_/bathocuproine (BCP)/Al. PC_71_BM/C_60_ is a double fullerene layer employed as an ETM to minimize the trap densities.^181^ Also, the perovskite solar cells with a p‐i‐n structure of ITO/PEDOT:PSS/(NH_4_)_3_Sb_2_I_9_/PC_61_BM/Al were synthesized to study the photovoltaic performance of (NH_4_)_3_Sb_2_I_9_ reporting a SPCE of 0.51%.^147^

The selection of charge extraction layers by modulating a desirable energy band alignment between the conduction band edge of CsSnI_3_ and LUMO of ETL is another feasible strategy. A *V*_OC_ of 0.55 V was reported for CsSnI_3_ perovskite solar device using p‐i‐n structure ITO/CuI/CsSnI_3_/indane‐C_60_‐bisadduct ICBA/BCP/Al architecture. Here, ICBA acted as an ETM.^159^ BCP is used as an interfacial material in between C_60_ derivatives and the metal electrodes. The FF was significantly improved by using electrode interfacial layer. In an inverted planar (p‐i‐n) FASnI_3_ perovskite device, C_60_ has been employed as an ETM for efficient electron extraction.^172^ A solution gel derived ZnO used as an ETL bilayer fabricated at <110 °C facilitates the improved energy level alignment and enhanced charge carrier extraction and a PCBM layer is used to reduce the hysteresis and enhance the perovskite thermal stability.

ZnO can be a potential candidate to replace TiO_2_ as an ETM layer without causing a marked effect on the performance of PSCs.^182^, ^183^ The doping of pure ZnO nanorods with Au/Al results in high electron mobility and high electron density.^184^ From SCAPS‐1D, the use of ZnO nanorods as an ETM and Cu_2_O as a HTM for MASnI_3_ perovskite devices has displayed the best performance among all the PSCs. Cu_2_O is a suitable HTM layer in PSCs due to its high hole mobility and low electron affinity. The device displayed a maximum SPCE for ZnO nanorods/MASnI_3_/Cu_2_O structure exhibiting a *J*_SC_ of 32.26 mA cm^−2^, *V*_OC_ of 0.85 V, FF of 0.74, and SPCE of 20.23%.^185^ The ETM used in a perovskite solar cell has a significant impact on the SPCE of the device when bl‐TiO_2_ layer in Ag_2_Bi_3_I_11_ is replaced by bl‐SnO_2_, there is a significant increase in current density from 1.33 mA cm^−2^ (TiO_2_) to 2.31 mA cm^−2^ (SnO_2_) attributed to better electron extraction by SnO_2_ ETM.^186^

## 7 Lead‐Free Perovskites

### 7.1 Tin‐Based Perovskites

Tin is the most suitable candidate for substitution of lead for lead‐free perovskite solar cell because of its similar valence electronic configuration as that of lead and approximate same ionic radius of Sn^2+^ (115 pm) as that of Pb^2+^ (119 pm). It has lower value of electronegativity Sn^2+^ (1.96) than that of Pb^2+^ (2.33).^237^ Tin‐based perovskites have optical bandgap of 1.2–1.6 eV most suitable for their use as light absorbers, large carrier mobilities, and low exciton binding energies of 18 meV.^105^, ^119^, ^187^ Tin‐based perovskites are represented by the general formula ASnX_3_ where A can be MA^+^, Fa^+^ or Cs^+^ cation, and X is a halogen anion.

Methylammonium tin halides MASnX_3_ have a direct bandgap of 1.20–1.35 eV, electron mobility of 2320 cm^2^ V^−1^ s^−1^, hole carrier mobility of 322 cm^2^ V^−1^ s^−1^,^105^, ^188^ and long charge carrier diffusion length of more than 500 nm.^117^ The first completely lead‐free Sn‐based perovskite MASnI_3_ was processed on a mesoporous TiO_2_ scaffold that achieved SPCE of 8.4% under 1 sun illumination in a highly inert atmosphere in a glove box with *V*_OC_ of 0.88 V, *J*_SC_ of 16.8 mA cm^−2^, and FF of 0.42 obtained from material having optical bandgap of 1.23 eV.^120^ A Sn‐based perovskite model with the novel architecture of glass/ZnO:Al/TiO_2_/Ch_3_NH_3_Sncl_3_/CuI/Au, devised by Mandadapu et al.,^189^ has been analyzed by using the solar cell capacitance simulator (SCAPS‐ID), with the predicted parameters such as thickness 0–6 µm, defect density of 10^14^ cm^−3^ of light absorber layer, and bandgap 1.3 eV. The model achieved a SPCE of 24.82%, *V*_OC_ of 1.04 V, *J*_SC_ of 3.50 mA cm^−2^, and FF of 0.78. The excellent results of this model clearly signify the enormous potential of Sn‐based perovskites for their efficient use in solar cells. Since then an extensive work has been carried out on preparation and characterization of Sn‐based perovskites material to examine their structural, optical, and charge transport abilities for efficient use as light absorber in perovskite solar cells.^188^ The perovskite solar cells with CH_3_NH_3_SnBr_3_ as light absorber reported a SPCE of 0.35% for coevaporation and 0.12% for sequential deposition method.^190^ The composition of a halide anion in mixed halide tin‐based perovskites has an influence on the photovoltaic performance exhibited by them. The mixed halide tin‐based perovskite MASnI_3−_ *_x_*Br*_x_* was investigated by altering the Br^−^/I^−^ ratio, it was reported that MASnBr_3_ as a light absorber displays more *V*_OC_ (0.88 V) and less *J*_SC_ (8.26 mA cm^−2^) in comparison to MASnIBr_2_ having *V*_OC_ (0.82 V) and *J*_SC_ (12.33 mA cm^−2^). Among all MASnI_3−_ *_x_*Br*_x_* perovskites, MASnIBr_2_ has the highest reported SPCE of 5.73% under stimulated full sunlight.^98^ Also, the position of band edge of mixed halides perovskites, MASnI_3−_ *_x_*Br*_x_* can be tuned from 954 nm (MASnI_3_) to 577 nm (MASnBr_3_) thus displaying a remarkable tunability of color. Also the mixed halides tin‐based perovskites MASnIBr_2−_ *_x_*Cl*_x_* has been fabricated for carbon‐based mesoscopic cells devoid of ETM and HTM layers by varying the composition of SnCl_2_/SnBr_2_. The solar device with MASnIBr_1.8_Cl_0.2_ achieved the best photovoltaic performance of 3.11% with a long‐term stability in air. The device exhibited excellent charge recombination and dielectric relaxation properties.^191^ However, tin‐based perovskites have low values of SPCE due to fast oxidation of divalent Sn^2+^ into a more stable state Sn^4+^ and easy formation of Sn vacancies due to small value of formation energy. As a consequence of it, there is a large charge carrier recombination and high levels of self p‐doping in Sn‐based perovskites films. Thus, a lot of research has been carried out to suppress oxidation of divalent Sn^2+^. SnF_2_ has been added to such films to inhibit the oxidation process so as to reduce the background carrier hole density by filling Sn vacancies. The entire fabrication process is carried out in an inert atmosphere in the glove box encapsulated by hot melt polymer film, a glass cover slide with sealed edges so as to avoid the oxidation of perovskite film on exposition to ambient air that could cause its fast degradation. Addition of 5‐ammonium valeric acid iodide to MASnI_3_ suppressed oxidation of Sn^2+^ for better stability of the perovskite device.^192^ Hypophosphorous acid was also used for reducing the oxidation of divalent Sn^2+^ thereby reducing the number of Sn vacancies and charge carrier density. As a consequence of it, there is enhancement in charge recombination lifetime by fourfold than that of the control device.^193^

A SPCE of 2.14% was reported for a perovskite solar cell having MASnI_3_ with SnF_2_ additive as a light absorber. The fabricated device achieved *V*_OC_ of 0.45 V, *J*_SC_ of 11.48 mA cm^−2^, and FF of 0.48 and has long lifetimes of 200 h under 1 sun degradation conditions.^194^ However, an excess of SnF_2_ deteriorates the perovskite film morphology and device performance indicating that SnF_2_ concentration must be kept very low; as a result, the background charge carrier density remains too large to achieve high efficiency, thus it becomes mandatory to explore new and more efficient ways to alleviate the background charge carrier density for better performance of the perovskite solar cell. It was also proposed that the fabrication of perovskite film must be carried out under a reducing vapor atmosphere to reduce the hole density in MASnI_3_ films by inhibiting the oxidation of Sn^2+^ during the fabrication process. The excess use of SnF_2_ induces the phase separation in perovskite films. As a result of exposure to excess SnF_2_, plate like aggregates are formed in the film, thus it was resolved to use nonsolvent dripping process along with SnF_2_ via the formation of SnF_2_–pyrazine complex. Pyrazine has a strong binding affinity to SnF_2_ thereby suppressing the phase separation induced by the excess use of SnF_2_.^195^ **Table** **4** shows some photovoltaic parameters of methylammonium tin halides.

**Table 4:** Photovoltaic parameters of methylammonium tin halides (MASnX_3_)

Light absorber	*E*_g_	*V*_OC_	*J*_SC_	FF	SPCE	Architecture	Ref.
MASnI_3_	1.23	0.88	16.8	0.42	6.4	FTO/c‐TiO_2_/mp‐TiO_2_/absorber/Spiro‐OMeTAD/Au	120
MASnI_3_	1.3	0.716	15.18	0.50	5.44	FTO/c‐TiO_2_/absorber/Spiro‐OMeTAD/Au	196
MASnI_3_+SnF_2_	1.3	0.32	21.4	0.46	3.15	FTO/c‐TiO_2_/mp‐TiO_2_/absorber/Au	162
MASnI_3_	1.3	0.38	12.1	0.36	1.7	ITO/PEDOT:PSS/poly‐TPD/absorber/C_60_/BCP/Ag	151
MASnI_3_	1.26	0.27	17.4	0.39	1.86	FTO/c‐TiO_2_/mp‐TiO_2_/absorber/PTAA/Au	197
MASnIBr_2_	1.75	0.82	12.33	0.57	5.73	FTO/c‐TiO_2_/mp‐TiO_2_/absorber/Spiro‐OMeTAD/Au	106
MASnBr_3_	2.15	0.88	8.26	0.59	4.27	FTO/c‐TiO_2_/mp‐TiO_2_/absorber/Spiro‐OMeTAD/Au	106
MASnBr_3_	2.2	0.50	4.27	0.49	1.12	FTO/c‐TiO_2_/mp‐TiO_2_/absorber/P3HT/Au	190
MASnBr_3_	1.41	0.20	4.5	0.36	0.3	ITO/PEDOT:PSS/absorber/PCBM/Bis‐C_60_/Ag	198
MASnI_3_+hydrazine vapor	1.3	0.38	19.9	0.51	3.80	FTO/c‐TiO_2_/mp‐TiO_2_/absorber/PTAA/Au	199
MASnI_3_+SnF_2_	–	0.45	11.8	0.40	2.14	FTO/PEDOT:PSS/absorber/C_60_/BCP/Ag	194
en[MASnI_3_]+SnF_2_	1.4	0.43	24.3	0.63	6.63	FTO/c‐TiO_2_/mp‐TiO_2_/absorber/PTAA/Au	142
MASnI_3_+SnF_2_		0.46	21.4	0.42	4.29	ITO/PEDOT:PSS/absorber/C_60_/BCP/Ag	150
(FA)_0.75_(MA)_0.25_SnI_3_	1.33	0.61	21.2	0.62	8.12	ITO/PEDOT:PSS/absorber/C_60_/BCP/Ag	150
(FA)_0.75_(MA)_0.5_SnI_3_	1.33	0.53	21.3	0.52	5.92	ITO/PEDOT:PSS/absorber/C_60_/BCP/Ag	150
FASnI_3_	1.36	0.48	21.3	0.64	6.60	ITO/PEDOT:PSS/absorber/C_60_/BCP/Ag	150
MASnIBr_2−_ *_x_*Cl*_x_* +0%SnCl_2_+100% SnBr_2_	1.81	0.31	13.37	0.52	2.18	Glass/FTO/TiO_2_/absorber/carbon	191
10% SnCl_2_+90% SnBr_2_	1.87	0.38	13.99	0.57	3.11	Glass/FTO/TiO_2_/absorber/carbon	191
25% SnCl_2_+75% SnBr_2_	1.97	0.35	11.06	0.47	1.87	Glass/FTO/TiO_2_/absorber/carbon	191
50% SnCl_2_+50% SnBr_2_	1.49	0.24	9.33	0.47	1.07	Glass/FTO/TiO_2_/absorber/carbon	191
75% SnCl_2_+25% SnBr_2_	1.36	0.19	13.34	0.32	0.81	Glass/FTO/TiO_2_/absorber/carbon	191
100% SnCl_2_+0% SnBr_2_	1.25	0.12	19.12	0.30	0.74	Glass/FTO/TiO_2_/absorber/carbon	191

Formamidinium tin iodide FASnI_3_ has a direct bandgap of 1.41 eV closer to the requisite bandgap value for use in perovskite solar cells and do possess a single stable phase over a broad temperature range up to 200 °C. Sn‐based perovskite FASnI_3_ is more stable than MASnI_3_ due to suppression of oxidation of Sn^2+^ by FA^+^.^188^, ^200^ FASnI_3_ is first used as light absorber in perovskite solar cell by Koh et al.^109^ The fabricated films displayed a SPCE of 2.1%, *J*_SC_ of 24.5 mA cm^−2^, *V*_OC_ of 0.2 V, and FF of 0.36. Additive SnF_2_ is incorporated into FASnI_3_ to suppress the oxidation of Sn^2+^ for better film morphology. A SPCE of 4.8% has been achieved by incorporating SnF_2_ in FASnI_3_ to form a complex with SnF_2_ thereby improving the morphology of the perovskite film and slowing down the rate of crystallization of perovskite thin film. Antisolvent process can play a very significant role in preparing the uniform and pinhole‐free compact thin film with the use of diethyl ether as an antisolvent. A significant SPCE of 6.22% has been achieved in FASnI_3_ by using antisolvent process under forward scan with a small photocurrent hysteresis and a highly reproducibility.^172^

SnF_2_–pyrazine complex has been used to further enhance the morphology of FASnI_3_ perovskite that achieved a SPCE of 4.8%, *V*_OC_ of 0.32 V, *J*_SC_ of 23.7 mA cm^−2^, and improved stability. The encapsulated FASnI_3_ films displayed a stable performance for over 100 d maintaining 98% of their initial efficiency.^195^Chlorobenzene is also used as an antisolvent for FASnI_3_ films. The A‐site cation in Sn‐based perovskite has a significant effect on photovoltaic performance. The use of diethylammonium (en) and FA^+^ at A‐site of tin‐based perovskite results in a wider bandgap and an improved stability of photovoltaic performance. The complex en [FASnI_3_] displayed a SPCE of 7.1% and retained 96% of its initial efficiency over 1000 h without encapsulation. Also, the addition of en at A‐site cation along with (FA/MA/Cs) SnI_3_ cannot reduce dimensionality of the perovskite to 2D.^192^

The first mixed design composition in tin‐based perovskite was reported on FA_1−_ *_x_*MA*_x_*SnBr_3_ with a cubic structure. The bandgap of the perovskite film was varied from 2.4 eV (*x* = 0) to 1.92 eV (*x* = 0.82) but the device displayed no photovoltaic performance.^201^

Another mixed A‐site cation perovskite (FA)*_x_*(MA)_1−_ *_x_*SnI_3_ has been investigated for its use as a light absorber in a perovskite solar cell with an invested structure. By tuning the ratio of FA and MA yields the different values of SPCE. A SPCE of 8.12% is achieved for (FA)_0.75_(MA)_0.25_SnI_3_ with *V*_OC_ of 0.61 V and bandgap of 1.33 eV. The high SPCE is attributed to the improved perovskite film morphology and reduced recombination process.^150^

Phenylethylammonium (PEA) is substituted at A‐site in tin‐based perovskite and pure 3D, 2D‐3D mixture and pure 2D perovskite are fabricated by tuning the ratio of PEA from 0 to 100% such that inside every two metal halide octahedral layers, there is bilayer of PEA. The resulting mixed cation perovskite is (PEA)_2_(FA)*_n_*_−1_Sn*_n_*I_3_ *_n_*_+1_ where *n* is the number of tin iodide layers in the structure unit (*n* ≥ 1). For *n* = 3, 4, optical bandgaps of 1.5 and 1.42 eV are achieved. The 2D tin perovskite displayed better moisture stability than their 3D counterparts. Incorporation of 20% PEA into FA leads to low‐dimensional mixed perovskite (PEA)_2_FA_8_Sn_9_I_28_. The as‐fabricated solar cells achieved a SPCE of 5.9% with *V*_OC_ of 0.55 V and *J*_SC_ of 14.4 mA cm^−2^.^156^

**Figure 11:**
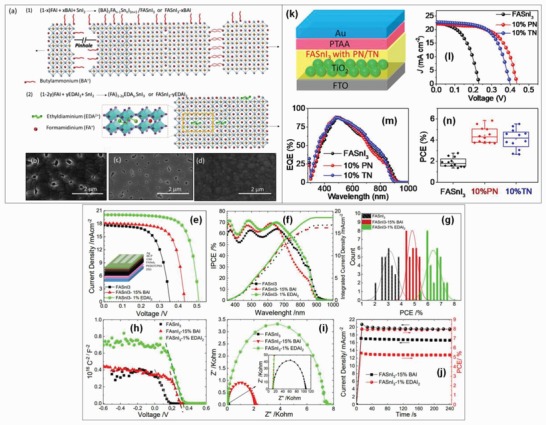
a) Schematic representations of perovskite crystals in the presence of BAI and EDAI_2_ additives; top‐view SEM images of b) pristine FASnI_3_, c) FASnI_3_‐BAI 15%, and d) FASnI_3_‐EDAI_2_ 1%; e) current–voltage curves, f) corresponding IPCE spectra with integrated current densities, g) histograms of 30 fresh cells fabricated under the same experimental conditions, h) Mott–Schottky plots, i) Nyquist plots obtained from electrochemical impedance spectra (EIS), and j) stabilized power‐conversion efficiencies and photocurrent densities of the FASnI_3_‐BAI 15%and FASnI_3_‐EDAI_2_ 1% devices for 240 s; Reproduced with permission.^203^ Copyright 2018, Royal Society of Chemistry. k) Device structure, l) *J*–*V* curves, m) EQE curves, and n) PCE statistics of the FASnI_3_ solar cells with and without 10% PN and 10% TN; Reproduced with permission.^206^ Copyright 2018, American Chemical Society.

The incorporation of phenylethylammonium iodide (PEAI) obtained by evaporating it at the bilateral interface of a FASnI_3_ film enhances the *V*_OC_ and FF of the mixed perovskite solar cell due to improvement in service coverage and formation of 2D‐3D bulk heterojunction structure whereas the presence of LiF at A‐site in tin‐based perovskite by evaporating it at the bilateral interface of FASnI_3_ film reduces the work function of PEDOT:PSS and aids in hole extracting at the ITO/PEDOT:PSS interfacial layer. A SPCE of 6.98% is achieved for LiF thickness of 5 nm in perovskite films with a *V*_OC_ of 0.47 V and FF of 0.74, respectively.^202^ The incorporation of BAI as an additive to the tin‐based perovskite film as a light absorber changes the orientation of the crystal growth thereby enhancing the connectivity of the crystal grains. The fabricated FASnI_3_ devices doped with 15% BAI exhibited a SPCE of 5.5% in contrast to the pristine FASnI_3_ films having 4% efficiency.^203^ Similarly, the doping of EDAI_2_ into FASnI_3_ films results in the removal of pinholes in the perovskite films, inhibition of oxidation of Sn^2+^ into Sn^4+^, and passivation of surface defect states. The 1% EDAI_2_‐doped FASnI_3_ perovskite films displayed an efficiency of 8.9% with a stability of over 1400 h with only slight degradation for more than 200 h in contrast to pristine FASnI_3_ films with a SPCE of 7.4% only. The high efficiency is attributed to improved perovskite film morphology and passivation of surface defects that enable better separation of charge carriers and suppression of oxidation of Sn^2+^ to Sn^4+^.^203^ **Figure** **11**a shows the schematic representations of perovskite crystals in the presence of BAI and EDAI_2_ additives; top‐view SEM images of (b) pristine FASnI_3_, (c) FASnI_3_‐BAI 15%, and (d) FASnI_3_‐EDAI_2_ 1%; (e) current–voltage curves, (f) corresponding IPCE spectra with integrated current densities, (g) histograms of 30 fresh cells fabricated under the same experimental conditions, (h) Mott–Schottky plots, (i) Nyquist plots obtained from electrochemical impedance spectra (EIS), and (j) stabilized power‐conversion efficiencies and photocurrent densities of the FASnI_3_‐BAI 15% and FASnI_3_‐EDAI2 1% devices for 240 s.^203^

Crystalline FASnI_3_ with the orthorhombic *a*‐axis out‐of‐plane direction was fabricated by mixing (0.08 m) 2D tin perovskite with (0.92 m) 3D tin perovskite in a planar p‐i‐n structure and achieved a SPCE of 9% with a *V*_OC_ of 0.25 V with more efficient collection of charge and stability in ambient air.^145^ The introduction of a low‐dimensional interlayer to the interface in a FASnI_3_ perovskite solar cell can improve the morphology of the film reducing the number of trap sites. As a result, the charge accumulation and recombination in the device is suppressed leading to a high SPCE of 7.05%.^204^

The bifunctional ammonium cations, 2‐hydroxyethyl ammonium OH(CH_2_)NH_3_^+^(HEA^+^), are incorporated into FASnI_3_ resulting in a mixed tin‐based perovskite HEA*_x_*FA_1−_ *_x_*SnI_3_ where *x* = 0–1 and can act as a light absorber in carbon‐based mesoscopic solar cells. As a consequence of incorporation of HEA^+^, the crystal lattice changed from orthorhombic to rhombohedra (*x* = 0.2–0.4). For *x* ≥ 0.6, a 3D vacant perovskite (HEA)*_x_*FA_1−_ *_x_*Sn_0.67_I_2.33_ with a tetragonal structure is formed. The light absorbers in this series are synthesized by employing mesoporous solar cells using one‐step drop‐cast (DC), two‐step solvent–solvent extraction (SE), and a solvent extraction by using ethylenediammonium diiodide (EDAI_2_) as an additive. The fabricated solar device HEA_0.4_FA_0.6_SnI_3_ displayed the photovoltaic parameters with *V*_OC_ of 0.371 V, *J*_SC_ of 18.52 mA cm^−2^, FF of 0.562, and a stable SPCE of 3.9% for a period of 340 h.^205^ The FASnI_3_ perovskite light absorbers incorporated with a diammonium cation such as propylenediammonium (PW) and trimethylenediammonium (TN) display better efficiency than the pristine FASnI_3_ solar cell. The FASnI_3_ light absorbers mixed with 10% TN and 10% PN displayed a SPCE of 5.53 and 5.58% with a better film morphology along with retaining their 3D perovskite structure.^206^ Figure 11 shows the (k) device structure, (l) *J*–V curves, (m) EQE curves, and (n) PCE statistics of the FASnI_3_ solar cells with and without 10% PN and 10% TN.^206^ The addition of bromide into FASnI_3_ crystal lattice reduces the p‐doping in the perovskite film by reducing the Sn vacancies thereby lowering the current density of the light absorbers. As a result, there is an enhancement in charge recombination lifetime that increases *V*_OC_ and FF of the devices having M‐TiO_2_ as electron transport layer. The fabricated devices achieved SPCE of 5.5% with high stability of encapsulated devices over 1000 h under continuous illumination including UV region.^167^ **Table** **5** shows some photovoltaic parameters of formamidinium tin halides.

**Table 5:** Photovoltaic parameters of formamidinium tin halides (FASnX_3_)

Light absorber	*E*_g_	*V*_OC_	*J*_SC_	FF	SPCE	Architecture	Ref.
FASnI_3_+SnF_2_	1.41	0.24	24.5	0.36	2.1	FTO/c‐TiO_2_/mp‐TiO_2_/absorber/Spiro‐OMeTAD/Au	195
FASnI_3_+SnF_2_ pyrazine	1.4	0.32	23.7	0.63	4.8	FTO/c‐TiO_2_/mp‐TiO_2_/absorber/Spiro‐OMeTAD/Au	109
FASnI_3_+diethyl ether	1.36	0.47	22.1	0.60	6.22	ITO/PEDOT:PSS/absorber/C_60_/BCP/Ag	172
FASnI_3_+SnF_2_	1.4	0.48	21.3	0.64	6.6	ITO/PEDOT:PSS/absorber/C_60_/BCP/Ag	150
FASnI_3_+SnF_2_	1.4	0.38	23.1	0.60	5.27	FTO/c‐TiO_2_/mpTiO_2_/ZnS/absorber/PTAA/Au	207
en[FASnI_3_]	1.5	0.48	22.5	0.66	7.14	FTO/c‐TiO_2_/mp‐TiO_2_/absorber/PTAA/Au	208
FA_0.25_MA_0.75_SnI_3_+SnF_2_	1.28	0.48	20.7	0.45	4.49	ITO/PEDOT:PSS/absorber/C_60_/BCP/Ag	150
FA_0.50_MA_0.50_SnI_3_+SnF_2_	1.33	0.53	21.3	0.52	5.92	ITO/PEDOT:PSS/absorber/C_60_/BCP/Ag	150
FA_0.75_MA_0.25_SnI_3_+SnF_2_	1.33	0.61	21.2	0.62	8.12	ITO/PEDOT:PSS/absorber/C_60_/BCP/Ag	150
FA_0.8_Cs_0.2_SnI_3_	1.41	0.24	16.05	0.35	1.4	ITO/PEDOT:PSS/absorber/PCBM/Bis‐C_60_/Ag	198
FASnI_3_	1.37	0.31	18.36	0.67	3.85	ITO/PEDOT:PSS/absorber/C_60_/BCP/Ag	202
FASnI_3_+50% PEAI	1.38	0.38	19.96	0.69	5.28	ITO/PEDOT:PSS/absorber/C_60_/BCP/Ag	202
(PEA)_2_(FA)_2_Sn_2_I_28_+SnF_2_	1.789	0.59	14.4	0.69	5.94	ITO/NiO*_x_*/absorber/PCBM/Al	156
(BA)_2_(MA)_3_Sn_4_I_13_+SnF_2_	1.42	0.229	24.1	0.45	2.53	FTO/c‐TiO_2_/mp‐TiO_2_/absorber/PTAA/Au	209
FASnI_3_+LiF (0 nm)	–	0.38	19.65	0.71	5.04	ITO/PEDOT:PSS/absorber/C_60_/BCP/Ag	202
FASnI_3_+LiF (3 nm)	–	0.42	19.83	0.73	5.96	ITO/PEDOT:PSS/absorber/C_60_/BCP/Ag	202
FASnI_3_+LiF (5 nm)	–	0.47	20.00	0.73	6.77	ITO/PEDOT:PSS/absorber/C_60_/BCP/Ag	202
FASnI_3_+LiF (8 nm)	–	0.45	16.66	0.65	4.71	ITO/PEDOT:PSS/absorber/C_60_/BCP/Ag	202
FASnI_3_	–	0.36	17.6	0.62	4.0	ITO/PEDOT:PSS/absorber/C_60_/BCP/Ag	203
FASnI_3_+15% (BAI)	1.40	0.44	18.0	0.69	5.5	ITO/PEDOT:PSS/absorber/C_60_/BCP/Ag	203
FASnI_3_+1% EDAI_2_	1.43	0.51	20.0	0.71	7.4	ITO/PEDOT:PSS/absorber/C_60_/BCP/Ag	203
FASnI_3_+2% EDAI_2_	1.44	0.58	21.3	0.71	9.6	ITO/PEDOT:PSS/absorber/C_60_/BCP/Ag	203
FASnI_3_	–	0.525	24.1	0.71	9.0	ITO/PEDOT:PSS/absorber/C_60_/BCP/Al	145
HEA_0.4_FA_0.6_SnI_3_ +3%EDAI_2_	–	0.371	18.52	0.562	3.9	–	205
FASnI_3_+10%TN	–	0.398	22.72	0.610	5.53	FTO/mp‐TiO_2_/absorber/PTAA/Au	206
FASnI_3_+10%PN	–	0.435	22.15	0.606	5.85	FTO/mp‐TiO_2_/absorber/PTAA/Au	206

Cesium tin iodide perovskite CsSnI_3_ possess a direct bandgap of 1.30 eV, a melting point of 435 °C indicating its better thermal stability and a 3D orthorhombic structure^110^, ^210^, ^211^ whereas cesium tin bromide perovskite CsSnBr_3_ has a bandgap of 1.7 eV.^102^ Cesium‐based tin perovskite has a high hole mobility of 585 cm^−1^ V^−1^ s^−1^, low exciton binding energy (180 meV) than MAPbI_3_.^119^, ^187^ The melt‐synthesized CsSnI_3_ ingots containing high‐quality large single crystal grains have been reported to have bulk carrier lifetime more than 6.6 ns, doping concentration of about 4.5 × 10^17^ cm^−3^, and minority carrier diffusion lengths approaching to 1 µm.^118^ A SPCE of 23% was predicted for optimized single crystal solar cells CsSnI_3_ highlighting their great potential for use in perovskite solar cell. The CsSnI_3_ was first used in a Schottky‐type perovskite solar cell consisting of simple layer architecture of ITO/CsSnI_3_/Au/Ti on a glass substrate that achieved an efficiency of 0.9%. A HTM‐free CsSnI_3_ perovskite solar cell with SnI_2_ as an additive displayed an efficiency of up to 2.76% with a *V*_OC_ of 0.43 V and FF of 0.39.^212^ The use of excess SnI_2_ as an additive in CsSnI_3_ not only suppress Sn^2+^ vacancies but also reduces p‐type conductivity thereby producing a SPCE of 4.8% in CsSnI_3_ perovskite solar cells.^213^ The thin films of CsSnIBr_3_ were fabricated with the addition of hypophosphorous acid (HPA) with thermal stability up to 473 K achieving a SPCE of 3% that last for over 77 d.^193^ The results of a computational study on mixed cesium perovskite Rb*_y_*Cs_1−_ *_y_*Sn (Br*_x_*I_3−_ *_x_*)_3_ as a light absorber have revealed that the substitution of Rb^+^ for Cs^+^ enhanced the quality of perovskite film and its practical applicability in perovskite solar cells.^214^ Another study on CsSnI_3_ and CsSnI_3−_ *_x_*Br*_x_* as light absorbers in n‐i‐p devices structure reported an efficiency of 2%. CsSnI_3_ has a small bandgap of 1.27 eV to a near‐infrared absorption onset to 950 nm and exhibited a high charge carrier density up to27.67 mA cm^−2^.^215^

An excess of SnCl_2_ and SnI_2_ to CsSnI_3_ perovskite films can have masked influence on both stability and SPCE of the corresponding cells reported to be of 3%. An extensive monitoring of oxidation of CsSnI_3_ in the air by using additives SnCl_2_, SnBr_2_, and SnI_2_ has been carried out to measure electronic, optical absorption spectrum with time and reported that it exhibits the highest stability by inhibiting the crystallization/decomposition.^163^, ^192^ The addition of SnF_2_ lowers the background charge carrier density by neutralizing traps.^214^, ^216^ The mesostructured CsSnI_3_ displayed a SPCE of 2.02% with the addition of 20% SnF_2_ as an additive. Also a spectral response of 950 nm is demonstrated with SnF_2_ addition. As a result, the concentration of the defect is reduced that further suppressed the background charge carrier density.^215^ The anionic substitution of Br^−^ in CsSnI_3−_ *_x_*Br*_x_* (0 ≤*x* ≤ 3) results in change in crystal structure from orthorhombic to cubic framework for CsSnBr_3_ enhancing the *V*_OC_ and *J*_SC_ as a result of decrease in tin vacancies and low charge carrier densities of 10^15^ cm^−3^.^217^ The carrier lifetime gets enhanced and the PL line width has reduced when the temperature decreases below 110 K due to the phase transition from orthorhombic to tetragonal phase in CsSnX_3_ that improved the solar cell performance.^218^

**Figure 12:**
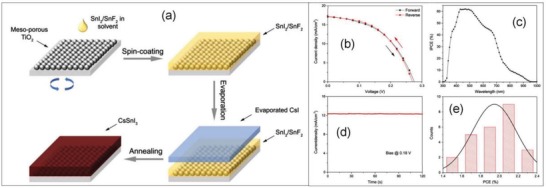
a) Schematic diagram for development of evaporation‐assisted solution (EAS) method using CsSnI_3_. b) *J*–*V* curves of the device by EAS method in both forward and reverse directions, c) IPCE spectrum of the optimized device (*V*_oc_ = 0.265 V, *J*_sc_ = 15.25 mA cm^−2^, FF = 46.05%, and PCE = 1.86%), d) steady‐state current density of champion device at a bias of 0.18 V, and d) PCE histogram of 25 tested devices. Reproduced with permission.^170^ Copyright 2018, Wiley‐VCH.

The evaporation method comprising of thermal evaporation with solution method has been used to produce smooth uniform dense pinhole‐free CsSnI_3_ films that achieved a SPCE of 1.86% with *V*_OC_ of 0.265 V, *J*_SC_ of 15.25 mA cm^−2^, and FF of 0.46, by using the architecture (FTO/bl‐TiO_2_/mp‐TiO_2_/absorber/Spiro‐OMeTAD).^102^The undesirable p‐doping of CsSnI_3_ perovskite films can be reduced by the addition of piperazine that can improve the morphology of the film as well as can alleviate the crystallization of excess SnI_2_ at the same time. With the use of piperazine as an additive, CsSnI_3_ perovskite devices displayed a SPCE of 3.83%.^219^ **Table** **6** shows some photovoltaic parameters of cesium‐based perovskites. **Figure** **12** shows schematic diagram along with the performance of CsSnI_3_‐based perovskite solar cell.^170^

**Table 6:** Photovoltaic parameters of cesium‐based perovskites

Light absorber	*E*_g_	*V*_OC_	*J*_SC_	FF	SPCE	Architecture	Ref.
CsSnI_3_+SnI_2_	1.3	0.38	25.71	0.49	4.81	FTO/c‐TiO_2_/mp‐TiO_2_/absorber/PTAA/Au	213
CsSnI_3_+SnI_2_	1.3	0.43	12.3	0.39	2.76	ITO/CuI/absorber/ICBA/BCP/Al	159
CsSnI_3_	1.3	0.52	10.2	0.62	3.31	ITO/NiO*_x_*/absorber/PCBM/Al	157
CsSnI_3_+SnCl_2_	1.3	0.50	9.89	0.68	3.56	ITO/absorber/PC_61_ BM/BCP/Al	163
CsSnI_3_+SnF_2_	1.27	0.20	22.7	0.29	1.66	FTO/TiO_2_/mp‐TiO_2_/absorber/Spiro‐OMeTAD/Au	217
CsSnI_3_+SnF_2_	1.25	0.17	30.8	0.34	1.83	FTO/c‐TiO_2_/mp‐TiO_2_/absorber/PTAA/Au	199
CsSnI_3_+SnF_2_	1.3	0.24	27.7	0.37	2.0	FTO/c‐TiO_2_/mp‐TiO_2_/absorber/m‐MTDATA/Au	215
CsSnI_3_	1.3	0.42	4.8	0.22	0.88	ITO/absorber/Au/Ti	212
CsSnBr_3_+SnF_2_	1.8	0.45	2.4	0.55	0.55	ITO/MoO_3_/absorber/C_60_/BCP/Ag	220
CsSnBr_3_+SnF_2_	1.75	0.41	3.99	0.58	0.95	ITO/mp‐TiO_2_/absorber/Spiro‐OMeTAD/Au	217
CsSnIBr_2_+SnF_2_	1.65	0.31	11.6	0.43	1.56	ITO/mp‐TiO_2_/absorber/Spiro‐OMeTAD/Au	217
CsSnIBr_2_+HPA‐SnF_2_	1.63	0.31	17.4	0.57	3.2	FTO/c‐TiO_2_/Al_2_O_3_/absorber/C	193
CsSnBr_3_+SnF_2_	1.79	0.37	14.0	0.59	3.04	FTO/c‐TiO_2_/mp‐TiO_2_/absorber/PTAA/Au	199
CsSnBr_3_+SnF_2_	1.75	0.42	9.1	0.58	2.1	FTO/c‐TiO_2_/mp‐TiO_2_/absorber/Spiro‐OMETAD/Au	102
CsSnI_2.9_Br_0.1_+SnF_2_	–	0.22	24.16	0.33	1.76	FTO/c‐TiO_2_/mp‐TiO_2_/absorber/Spiro‐OMETAD/Au	217
CsSnI_3_	–	0.265	15.25	0.46	1.86	FTO/bl‐TiO_2_/mp‐TiO_2_/absorber/Spiro‐OMETAD/Au	170

Tin in +4 oxidation state shows more air and moisture stability with enhanced photovoltaic properties. To combat the challenge of oxidation of Sn^2+^ to Sn^4+^, tin‐based perovskite structures like A_2_SnX_6_ are investigated for their use in a perovskite solar cell.^105^, ^221^, ^222^ Tin‐based Cs_2_SnI_6_ as a light absorber has reported a SPCE of almost 1%. These perovskites have been investigated for their use as hole transport material in solar cells.^223^ Cs_2_SnI_3_Br_3_ 222 and Cs_2_SnI_6_ 224 as light absorbers in solid‐state dye‐sensitized solar cells have displayed an efficiency of 7.8%^224^ by using classical dyes as a light absorber. Cs_2_SnI_6_ used as a hole transport material in solid‐state DSSCs reported an efficiency close to 8.6% in air.^224^ Cs_2_SnI_6_ has a direct bandgap of 1.3–1.6 eV, high absorption coefficient, high electron carrier concentration of the order of 1 × 10^14^ cm^−3^, electron mobility of 310 cm^2^ V^−1^ s^−1^, and better stability in air with moisture than that of CsSnI_3_ as Sn^4+^ is chemically more stable than Sn^2+^.^105^, ^216^, ^225^ The cesium‐based perovskite Cs_2_SnI_6_ as a light absorber was first studied in 2016 that reported an efficiency of 1%.^216^, ^223^ Cs_2_SnI_6_ do possess defects of iodide vacancies and interstitial Sn atoms that give rise to the intrinsic n‐type behavior completely opposite to p‐type behavior in CsSnI_3_. Optimization of thickness of perovskite light absorption layers leads to spontaneous oxidation conversion of unstable B‐ϒCsSnI_3_ to air stable Cs_2_SnI_6_ that has bandgap of 1.48 eV and a high absorption coefficient of 10^5^cm^−1^.^216^ The bandgap of A_2_SnX_6_ perovskite depends upon the composition of halide anion. With increase in bromide composition CsSnI_6−_ *_x_*Br*_x_*, the bandgap can be tuned from 1.3 to 2.9 eV and the color of the film changes from dark brown to brown red then to yellow. Cs_2_SnI_4_Br_2_ reported an efficiency of 2.03% highest among all the fabricated compositions. The fabrication of all the reported composition was done in ambient air without the use of any additive and the perovskite film exhibited thermal stability.^226^ The polycrystalline films of (MA)_2_SnI_6_ have been proposed by using thermal evaporation method having a direct bandgap of 1.81 eV with a strong absorption coefficient of 7 × 10^4^ cm^−1^, carrier concentration of 2 × 10^15^ cm^−3^, and electron mobility of ≈3 cm^2^ V^−1^ s^−1^.^129^ **Table** **7** shows some photovoltaic parameters of cesium‐based perovskites.

**Table 7:** Photovoltaic parameters of tin‐based perovskites A_2_SnX_6_

Light absorber	*E*_g_	*J*_SC_	*V*_OC_	FF	SPCE	Architecture	Ref.
Cs_2_SnI_6_	1.48	5.41	0.51	0.35	0.96	FTO/TiO_2_/absorber/P3HT/Ag	216
Cs_2_SnI_6_	1.30	6.75	0.37	0.59	1.47	FTO/bl‐TiO_2_/2% Sn‐TiO_2_/absorber/Cs_2_SnI_6_HTM/LPAH/FTO	226
Cs_2_SnI_5_Br	1.38	6.58	0.44	0.55	1.60	FTO/bl‐TiO_2_/2% Sn‐TiO_2_/absorber/Cs_2_SnI_6_HTM/LPAH/FTO	226
Cs_2_SnI_4_Br_2_	1.40	6.23	0.56	0.57	2.03	FTO/bl‐TiO_2_/2% Sn‐TiO_2_/absorber/Cs_2_SnI_6_HTM/LPAH/FTO	226
Cs_2_SnI_2_Br_4_	1.63	3.41	0.58	0.54	1.08	FTO/bl‐TiO_2_/2% Sn‐TiO_2_/absorber/Cs_2_SnI_6_HTM/LPAH/FTO	226
Cs_2_SnIBr_5_	2.36	0.01	0.57	0.37	0.002	FTO/bl‐TiO_2_/2% Sn‐TiO_2_/absorber/Cs_2_SnI_6_HTM/LPAH/FTO	226
Cs_2_SnBr_6_	2.85	–	–	–	–	FTO/bl‐TiO_2_/2% Sn‐TiO_2_/absorber/Cs_2_SnI_6_HTM/LPAH/FTO	226

### 7.2 Germanium‐Based Perovskites

Germanium is another candidate for substitution of lead for lead‐free perovskite solar cells because of its valence electronic configuration as that of Pb^2+^. Ge^2+^ has a small ionic radius (73 pm) as compared to that of divalent metal cation Pb^2+^ (119 pm) and Sn (110 pm). Ge^2+^ is low in toxicity than Pb^2+^.^227^ However, germanium is prone to oxidation than tin. It has a value of electronegativity (2.1) as compared to Pb (3.2) and Sn (1.96). Methyl ammonium germanium halides MAGeX_3_ are the most potential candidate for perovskites solar cells as Goldschmidt tolerance factor for MAGeX_3_[X‐Cl, Br, I] has value of 1.005,0.988, and 0.965, respectively, that is close to the optimum range 0.99 < *t* < 1.03 for a material to form a stable 3D perovskite structure. MAGeI_3_ has an optical bandgap 1.63 eV which is greater in magnitude than that of MAPbI_3_(1.55 eV) and MASnI_3_(1.30), excellent hole and electron conducting behavior and better stability in air as compared to MaPbI_3_.^217^ However, Ge^2+^ cation being smaller in size (73 pm) deviates from its regular [GeI_6_] octahedral center as it replaces cation of much larger ionic radius as that of Pb^2+^ (119 pm) and Sn^2+^ (110 pm).^228^ As a consequence, it forms three short Ge—I bonds (2.73–2.77 Å)^228^ and three long in Ge—I bonds (3.26–3.58 Å). The Ge‐based perovskites have been extensively studied by carrying computational work based on density functional theory (DFT).^229^, ^230^ The size of constituent halide ion has a remarkable effect on the bandgap of Ge‐based perovskite. The DFT calculations of bandgap values of CsGeX_3_[X‐Cl, Br, I] showed the decreasing trend of 3.67, 2.32, and 1.53 eV, respectively.^231^ Similar trend is noticed in MAGeI_3_[X‐Cl, Br, I] whose DFT calculations reveal that with the increase in size of halide anion, the bandgaps have decreasing values of 3.7, 2.81, and 1.61 eV.^230^ The cation at A‐site also plays a pivotal role for the size of bandgap of AGeI_3_.^144^, ^230^ Bandgaps show an increasing trend when small Cs^+^ cation(1.6 eV) is replaced by a larger counterpart such as CH_3_NH_3_^+^ (1.9 eV) and CH(NH_2_)^+^ (2.2 eV), acetamidinium (2.5 eV), trimethylammonium (2.8 eV), guanidinium (2.7 eV), and isopropyl ammonium (2.7 eV).^229^

A study of Ge‐based perovskite AGeX_3_ (A‐Cs^+^, CH_3_NH_3_^+^, HC(NH_2_)_2_^+^) reported the estimated values of optical bandgap derived from tauc plot for CsGeI_3_ (1.63 eV), MAGeI_3_ (2.0 eV), and FAGeI_3_ (2.3 eV).^144^ The replacement of Cs with MA and FA decreases the valence band level as evident from measured value of valence band of CsGeI_3_, MAGeI_3_, and FAGeI_3_ that has the value of −5.10, −5.2, and −5.5 eV, respectively, by photoemission spectroscopy in air.^144^ CsGeI_3_ displays a higher stability up to 850 °C in contrast to up to ≈250 °C stability shown by MAGeI_3_ and FAGeI_3_. Ge‐based perovskite solar cells have two values of *V*_OC_ due to its oxidation into Ge^4+^ during the fabrication process. The poor quality of FAGeI_3_ films results in loss of photoconductivity in them.^144^ The small A‐site cations like Cs^+^, CH_3_NH_3_^+^, and HC(NH_2_)_2_^+^ in AGeX_3_ lead to 3D structure framework based on corner‐sharing octahedral and the perovskite materials do exhibit direct bandgaps whereas large A‐size cations lead to distortion of the crystal structure. As a result, 1D chain like perovskite structures are formed having indirect bandgaps.^144^, ^229^ The introduction of bromide ions into MAGeI_3_ perovskites enhances not only photovoltaic performance but also stability to a slight extent.^232^ The substitution of 10% of the iodide content by bromide results in MAGeI_2.7_Br_0.3_ perovskite that reported a SPCE of 0.57% as a light absorber in solar cells fabricated with planar p‐i‐n architecture having PEDOTS:PSS as HTM and PC_70_BM as ETM.^232^

**Figure 13:**
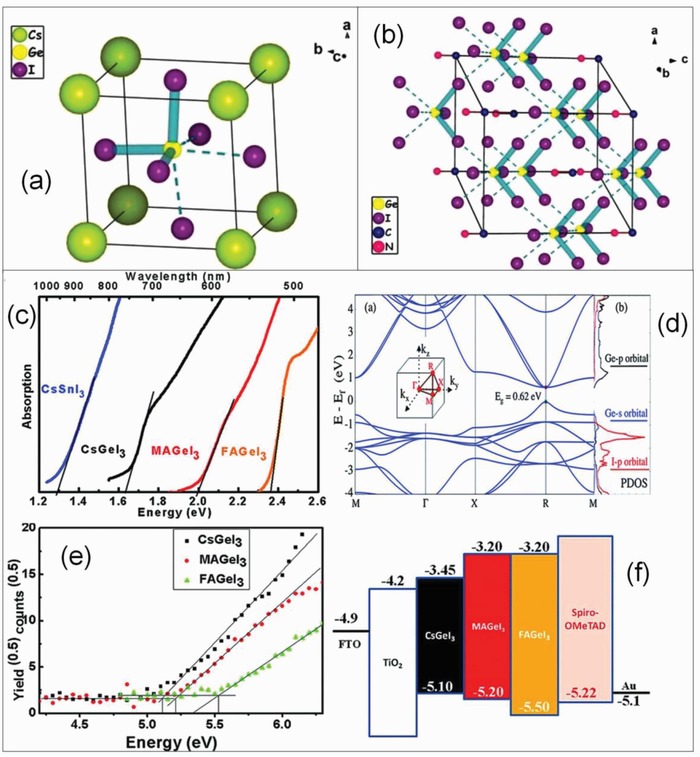
Schematic diagram for the unit cell of a) CsGeI_3_ and b) MAGeI_3_; Reproduced with permission.^229^ Copyright 2015, American Chemical Society. c) Optical absorption spectrum of CsGeI_3_, MAGeI_3_, and FAGeI_3_, in comparison with CsSnI_3_. d) Calculated band structure and projected density of states of CsGeI_3_. The energy of the highest occupied state is set to 0 eV. e) Photoelectron spectroscopy in air (PESA) of powder samples and f) schematic energy level diagram of CsGeI_3_, MAGeI_3_, and FAGeI_3_; Reproduced with permission.^144^ Copyright 2012, Royal Society of Chemistry.

The mixed Ge‐based perovskite RbSn_0.5_Ge_0.5_I_3_ displays a direct optical bandgap in the range of 0.9–1.6 eV with sufficient optical absorption spectrum comparable to MAPX_3_ perovskites. The material exhibited favorable effective masses for higher carrier mobility and good stability in water.^233^ A 2D perovskite (C_6_H_5_(CH_2_)_2_NH_3_)_2_GeI_4_ [(PEA)_2_ GeI_4_] consisting of inorganic germanium iodide planes separated by organic PEAI layers has a direct bandgap of 2.12 eV that is very close to the value 2.17 eV obtained through DFT calculations. The perovskite material exhibits luminescence at room temperature with a medium lifetime and is a potential candidate for PV applications. The 2D (PEA)_2_GeI_4_ shows more stability in air than 3D MAGeI_3_ that is attributed to the presence of a hydrophobic organic long chain.^234^ On the basis of DFT calculations, one more 2D Ruddlesden–Popper hybrid organic–inorganic perovskite BA_2_MA*_n_*_−1_MnI_3_ *_n_*_+1_ [M = Sn or Ge, *n* = 2–4] has been reported that has suitable excitonic and optical light absorbing properties for application in lead‐free perovskites. Moreover, 2D Ge‐based perovskites have enhanced thermodynamic stability in comparison to their 3D counterparts that enables 2D Ge‐based perovskites with a thickness of a few tens of unit cells to be used as light absorbers in perovskite solar cell.^235^ **Table** **8** shows some photovoltaic parameters of Ge‐based perovskites. **Figure** **13** shows the crystal structure, band diagram, and the *I–V* characteristics of Ge‐based perovskites in a solar cell (a) CsGeI_3_ and (b) MAGeI_3_,^229^ (c) optical absorption spectrum of CsGeI_3_, MAGeI_3_, and FAGeI_3_, in comparison with CsSnI_3_, and (d) calculated band structure and projected density of states of CsGeI_3_. The energy of the highest occupied state is set to 0 eV. (e) Photoelectron spectroscopy in air (PESA) of powder samples and (f) schematic energy level diagram of CsGeI_3_, MAGeI_3_, and FAGeI.^144^

**Table 8:** Ge‐based perovskites and their photovoltaic parameters

Light absorber	*E*_g_	*V*_OC_	*J*_SC_	FF	SPCE	Architecture	Ref.
MAGeI_3_	2.0	0.15	4.0	0.30	0.2	FTO/c‐TiO_2_/mp‐TiO_2_/absorber/Spiro‐OMeTAD/Au	144
CsGeI_3_	1.63	0.07	5.7	0.27	0.11	FTO/c‐TiO_2_/mp‐TiO_2_/absorber/Spiro‐OMeTAD/Au	144
CsGeI_3_	–	0.57	10.49	0.53	3.2	FTO/mp‐TiO_2_/CsGeI_3_/P3HT Au	236

### 7.3 Bismuth‐Based Perovskites

Bismuth can form +3 ions with similar valence electronic configuration as that of Pb^2+^, having ionic radius (103 pm) in comparison to divalent Pb^2+^ (119 pm) and Sn^2+^ (110 pm). The value of electronegativity of bismuth is 2.02 in comparison to that of Pb (2.33) and Sn (1.96).^237^ Bismuth‐based perovskites are represented with a general formula A_3_Bi_2_X_9_, where A can be MA, Cs, NH_3_, Ag. These materials have attracted large attention due to their low toxic nature.^238^ They can have 0D dimer, 1D chain like, 2D layered, or 3D double perovskite elpasolite frameworks,^238^ containing A‐site cations such as MA^+^, Cs^+^, Rb^+^, K^+^, guanidinium, cyclohexylammonium, imidazolium to form a 0D dimer perovskite structure.^143^ Bismuth‐based methylammonium single crystal MABi_2_I_9_ (MBI) shows a regular hexagon shape with a diameter ranging from 100 to 200 nm. The MBI crystals exhibit a dark red color with an optical bandgap of ≈2.11 eV. The 0D MBI consists of face‐sharing bi‐octahedral [Bi_2_I_9_] clusters surrounded by MA^+^ cations. The fabricated solar cells using MBI as light absorbing layer reported an efficiency of 0.08% with *J*_SC_ (≈0.36 mA cm^−2^), *V*_OC_ of 0.51 V, and FF of 0.44. The photovoltaic performance was enhanced by using thick mesoporous TiO_2_ layer (1.8 µm) to *V*_OC_ of 0.51 V, *J*_SC_ of 1.16 mA cm^−2^, FF of 0.46, and SPCE of 0.19%.^127^

The positive Hall coefficient of MBI film reveals p‐type charge carrier with carrier concentration of 10^15^–10^16^ cm^−3^ for solution‐processed MBI films. MBI films have got excellent stability against exposure to humidity level of 50% and ambient air at room temperature for 40 d.^127^ The first study on bismuth‐based perovskite (MA)_3_Bi_2_I_9_ as a light absorber was reported by preparing simple (MA)_3_Bi_2_I_9_ perovskite and mixed (MA)_3_Bi_2_I_9−_ *_x_*Cl*_x_* perovskite thin films with a hexagonal crystalline phase. The mesostructured solar cells displayed a better SPCE of 0.12%, *V*_OC_ of 0.68 V, *J*_SC_ of 0.52 mA cm^−2^, and FF of 0.33 as compared to (MA)_3_Bi_2_I_9−_ *_x_*Cl*_x_* displaying SPCE of 0.003, *V*_OC_ of 0.04 V, *J*_SC_ of 0.18 mA cm^−3^, and FF of 0.38. Also the substitution of iodine with chloride in (MA)_3_Bi_2_I_9−_ *_x_*Cl*_x_* shifted the bandgap from 2.1 to 2.4 eV.^111^ An efficiency of 0.42% is achieved by using a mesoporous TiO_2_ substrate for fabricating a (MA)_3_Bi_2_I_9_ perovskite film with *V*_OC_ of 0.67 V, *J*_SC_ of 1.0 mA cm^−3^, and FF of 0.62.^240^ MA_3_Bi_2_I_9_ films fabricated by evaporation‐spin‐coating process produced better quality films which produced SPCE of 0.39% in an inverted planar device with a *V*_OC_ of 0.83 V, *J*_SC_ of 1.39 mA cm^−2^, and FF of 0.34.^241^ The gas‐assisted deposition method enhances the morphology of active light absorber layer. The fabricated (MA)_3_Bi_2_I_9_ light absorber layer by gas‐assisted deposition process reported an enhanced value of SPCE of 0.08% and *V*_OC_ of 0.686 V.^242^ The solvent annealing in (MA)_3_Bi_2_I_9_ films enhances its electrical conductivity. The DMF‐induced solvent annealing impacts the charge transport through the films.^243^ The morphology of (MA)_3_Bi_2_I_9_ perovskite film can also be enhanced by incorporating a small amount of N‐methyl‐2 pyrrolidone (NMP) into the MBI‐DMF solution. The addition of various concentration of NMP into the precursor solution not only controls the rate of crystallization but also enhanced SPCE to a value of 0.31% and stability for 30 d in a relative humidity of 50–60%.^244^ The optical measurement of solution‐processed perovskite film (MA)_3_Bi_2_I_9_ fabricated by spin‐coating process showed a strong absorption band around 500 nm on further heating. The devices made on anatase TiO_2_ mesoporous layer exhibited a current density of 0.8 mA cm^−2^ whereas those fabricated by using brookite TiO_2_ layer do not display any current density.^174^ There is considerable effect of solvent treatment and substrate temperature on the morphology and structure of bismuth‐based perovskite films of MA_3_Bi_2_I_9_. The electron transport layer of fluorinated perylene diimide (FPD) treated by solvent vapor annealing with chloroform reported an efficiency of 0.06% for substrate temperature at 75 °C. The perovskite solar cell MA_3_Bi_2_I_9_ exhibited a small degradation after 17 d storage in ambient air conditions.^245^

The concentration of perovskite solution also impacts the morphology and photovoltaic performance of spin‐coated MA_3_Bi_2_I_9_ solar cells. The fabricated cells displayed an efficiency of *V*_OC_ (0.73 V) and efficiency of 0.17% after 48 h in air. The solar cells exhibited 56% of peak efficiency and 84% of open‐circuit voltage even after 300 h exposure in ambient air.^246^ The vapor‐assisted solution process (VASP) applied to BiI_3_ films by exposing them to CH_3_NH_3_I vapors results in enhancement of film morphology, efficiency, and stability in ambient air. The solar cell fabricated using pure BiI_3_ films and CH_3_NH_3_ vapors on mesoporous TiO_2_ substrate displayed high SPCE up to 3.17% attributed to better morphology, improved device composition, reduced metallic content, and suitable optoelectronic properties of the fabricated material that maintained a stability for 60 d with only 0.1% drop in efficiency.^247^ The wide bandgap of lead‐free perovskite devices (*E*_g_ > 1.9 eV) can be engineered to a narrow bandgap by incorporating triiodide into (4‐methyl piperidinium)_3_ Bi_2_I_9_ (MP‐Bi_2_I_9_) that resulted in 0D perovskite (MP‐T‐BiI_6_) (4‐methyl piperidinium)_4_I_3_BiI_6_. MP‐T‐BiI_6_ displayed a narrow bandgap of 1.58 eV comparable to 1.5 eV of MAPbI_3_, hole mobility (≈12.8 cm^2^ V^−1^ s^−1^), and charge trap density (≈1.13 × 10^10^ cm^−3^). The narrow bandgap signifies its potential to be used as an effective light absorber in perovskite solar cells.^248^ The solvent engineering method can be applied at bismuth‐based perovskite to produce pinhole‐free films of MA_3_Bi_2_I_9_, Cs_3_BiI_9_, or (MA)_3_Bi_2_I_9_. The fabricated MA_3_Bi_2_I_9_ films are most suitable for efficient and stable perovskite solar cells than the pristine MA_3_Bi_2_I_9_ films with pinholes.^249^ An enhanced open‐circuit voltage of 0.84 V is obtained in (MA)_3_Bi_2_I_9_ perovskite by using ethanol as solvent.^250^ The film quality of (MA)_3_Bi_2_I_9_ can be enhanced by high‐vacuum BiI_3_ deposition and low‐vacuum transformation of BiI_3_ to (MA)_3_Bi_2_I_9_. The fabricated perovskite solar cells exhibited a SPCE of 1.64%, *J*_SC_ of 2.95 mA cm^−2^, *V*_OC_ of 0.81 V, FF of 0.69, and long stability.^251^ 0D Cs_3_Bi_2_I_9_ perovskite films in mesostructured perovskite solar cells exhibited a SPCE of 1.09% with a *V*_OC_ of 0.81 V, *J*_SC_ of 2.95 mA cm^−2^, and FF of 0.69^111^ with a bandgap of 2.2 eV.

1D iodobismuthates consisting of 1D chain like BiI_4_^−^ anions with edge‐sharing BiI_6_ octahedra have been prepared from aqueous solutions. The four reported compounds LiBiI_4_·5H_2_O, MgBi_2_I_8_·8H_2_O, MnBi_2_I_8_·8H_2_O, and KBiI_4_·H_2_0 have direct bandgaps of 1.70–1.76 eV and can be used as potential light absorber.^252^ The 1,6 hexadiammonium bismuth halide perovskite (HDABiI_5_) showing 1D chain like structure is prepared by solution method. The (HDABiI_5_) m‐str. perovskite displayed a SPCE of 0.027%, *V*_OC_ of 0.40 V, *J*_SC_ of 0.12 mA cm^−2^, and FF of 0.43 with an optical bandgap of 2.05 eV.^253^ K_3_Bi_2_I_9_ and Rb_3_Bi_2_I_9_ are 2D layered defect perovskites prepared by solution method or solid‐state reactions. K_3_Bi_2_I_9_ and Rb_3_Bi_2_I_9_ have a direct bandgap of 2.1 eV.^239^ The perovskite film CsBi_3_I_10_ has a layered 2D structure as evident from X‐ray diffraction (XRD) pattern with a bandgap of 1.77 eV which is smaller than the bandgap of Cs_3_Bi_2_I_9_ (2.03 eV), absorption coefficient 1.4 × 10^5^ cm^−1^. The perovskite solar cell with CsBi_3_I_10_ achieved a photocurrent up to 700 nm leading to better scope for use in solar cells.^143^ The Cs_3_Bi_2_I_9_ films have a better film morphology and pinhole‐free layers. The CsBi_3_I_10_ films as a light absorber in mesostructured solar cells displayed a SPCE of 0.4% whereas Cs_3_Bi_2_I_9_ solar cells have displayed a SPCE of 0.02% only in same device architecture.^143^ Another 2D layered perovskite is MA_3_Bi_2_I_9_ which is prepared from solution and has a bandgap of 2.04 eV which is smaller than that of k_3_Bi_2_I_9_ and Rb_3_Bi_2_I_9_ (2.1 eV).^254^, ^255^ Bismuth‐based 3D double perovskite has been proposed with a chemical formula A_2_^I^B^I^Bi^III^X_6_ to maintain a charge neutrality of the perovskite material. The double perovskites like Cs_2_AgBiX_6_ (Br, Cl)^254^, ^255^, ^256^, ^257^ and (MA)_2_KBiCl_6_ 258 have been synthesized by using a solution method. It has been reported that Cs_2_AgBiBr_6_ 254 and Cs_2_AgBiCl_6_ have an indirect bandgap of 2.19 and 2.77 eV. (MA)_2_KBiCl_6_ has too large bandgap of 3.04 eV to be suitable for use in perovskite solar cells.^258^ The DFT calculations have revealed that double perovskite (MA)_2_TlBiI_6_ has a bandgap of 2.00 V potential to be used as a lead‐free perovskite material due to similar property as that of MAPbI_3_ but Tl is toxic in nature.^259^ The bimetal iodide thin films AgBi_2_I_7_ show a SPCE of 1.22%, *V*_OC_ of 0.56 V, *J*_SC_ of 3.30 mA cm^−2^, and FF of 1.87 with a better stability under ambient conditions.^260^ Using first principle calculations, a 3D double perovskite family has been revealed with optical bandgaps in the visible range and low carrier effective masses. The members of this family Cs_2_CuBiX_6_, Cs_2_AgBiX_6_, and CsAuBiX_6_ have optical bandgaps in the range of 1.3–2.0, 1.6–2.7, and 0.5–1.6 eV, respectively.^261^

**Figure 14:**
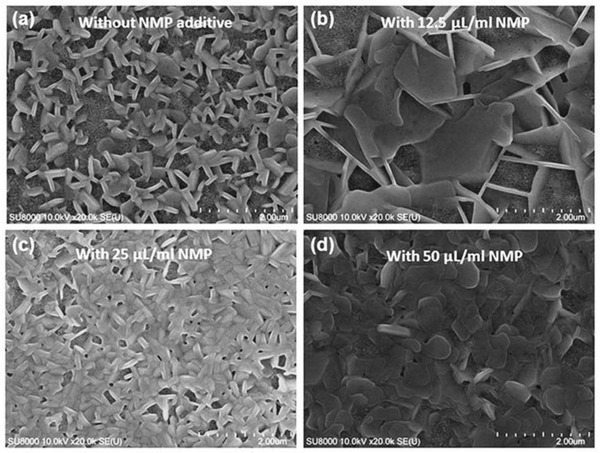
SEM images of (MA)_3_Bi_2_I_9_ without and with different concentration of NMP additives. Reproduced with permission.^244^ Copyright 2017, Royal Society of Chemistry.

The bismuth‐based perovskite solar cells Cs_3_Bi_2_I_9_ fabricated by glass/FTO/TiO_2_/Cs_3_Bi_2_I_9_/PTAA/Au architecture displayed an efficiency of 8%.The perovskite film of Cs_3_Bi_2_I_9_ exhibited a pure crystalline phase and excellent thermal stability. The encapsulated perovskite cell displayed constant efficiency for more than 500 h as light absorber at 65 °C with humidity at 60–70% level. The stability is attributed to the large size of bismuth‐based perovskite structure than lead‐based perovskite structure.^141^ The lattice compression of 0D perovskite Cs_3_Bi_2_I_9_ results in change in their structural, optical, and electrical properties. It is a result of lattice compression that there is an increase in exciton binding energy leading to an enhancement in emission under mild pressure. Bi—I bond contraction causes bandgap narrowing and an increase in metal halide orbital overlapping resulting from decrease in bridging Bi—I—Bi angle. These changes are reversible on decomposition. There is a semiconductor to conductor transition at ≈28 GPa due to decrease in resistance thus leading to metallization of Cs_3_Bi_2_I_9_.^262^ The high‐quality polycrystalline films of Cs_3_Bi_2_I_9_, Rb_3_Bi_2_I_9_, and AgBi_2_I_7_ can be fabricated by two‐step Co‐evaporation process involving two square evaporation of CsI, RbI or AgI and BiI_3_ and further annealing under BiI_3_ vapors producing films with better pinhole‐free morphology of films with average grain size >200 nm.^263^ Bismuth‐based perovskite films can be further engineered to produce pinhole‐free films of MA_3_Bi_2_I_9_ and Cs_3_Bi_2_I_9_. The MA_3_Bi_3_I_9_ films are more suitable for perovskite solar cells than the pristine.^14^ **Table** **9** shows some photovoltaic parameters of bismuth‐based perovskites. **Figure** **14** shows the SEM images of (MA)_3_Bi_2_I_9_ without and with different concentration of NMP additives.^244^

**Table 9:** PV parameters of bismuth‐based perovskites

Light absorber	*E*_g_	*V*_OC_	*J*_SC_	FF	SPCE	Architecture	Ref.
MA_3_Bi_2_I_9_	2.11	0.35	1.16	0.46	0.19	FTO/TiO_2_/mp‐TiO_2_/absorber/P3HT/Au	127
MA_3_Bi_2_I_9_	2.1	0.68	0.52	0.33	0.12	FTO/TiO_2_/mp‐TiO_2_/absorber/Spiro‐OMeTAD/Ag	111
MA_3_Bi_2_I_9_	2.26	0.72	0.49	0.31	0.11	FTO/TiO_2_/absorber/Spiro‐OMeTAD/Au	128
MA_3_Bi_2_I_9_	2.1	0.68	0.37	0.32	0.08	FTO/TiO_2_/absorber/Spiro‐OMeTAD/Au	242
MA_3_Bi_2_I_9_	–	0.56	0.83	0.49	0.26	FTO/TiO_2_/mp‐TiO_2_/absorber/Spiro‐OMeTAD/Au	173
MA_3_Bi_2_I_9_	–	0.51	0.94	0.61	0.31	FTO/TiO_2_/mp‐TiO_2_/absorber/Spiro‐OMeTAD/Au	244
MA_3_Bi_2_I_9_	2.1	0.65	1.10	0.50	0.36	FTO/TiO_2_/mp‐TiO_2_/absorber/Spiro‐OMeTAD/Au	264
MA_3_Bi_2_I_9_	2.22	0.83	1.39	0.34	0.39	ITO/PEDOT:PSS/absorber/C_60_/BCP/Ag	242
MA_3_Bi_2_I_9_	2.1	0.67	1.0	0.62	0.42	ITO/TiO_2_/mp‐TiO_2_/absorber/Spiro‐OMeTAD/Ag	240
MA_3_Bi_2_I_9_	2.9	0.66	0.22	0.49	0.07	ITO/PEDOT:PSS/absorber/PCBM/Ca/Al	143
MA_3_Bi_2_I_9−_ *_x_*Cl*_x_*	2.4	0.04	0.18	0.38	0.003	FTO/TiO_2_/mp‐TiO_2_/absorber/Spiro‐OMeTAD/Au	111
(MA_3_Bi_2_I_9_)_0.2_(BIi_3_)_0.8_	–	0.57	0.27	0.50	0.08	FTO/TiO_2_/mp‐TiO_2_/absorber/PTAA/PIDT‐DFBT/Ag	128
HDABiI_5_	–	0.40	0.12	0.43	0.027	FTO/c‐TiO_2_/HDABiI_5_/mp‐TiO_2_/Spiro‐OMeTAD/Au	253
Cs_3_Bi_2_I_9_	2.1	–	–	–	8.0	Glass/FTO/TiO_2_/Cs_3_Bi_2_I_9_/PTAA/Au	141
MA_3_Bi_2_I_9_+FPDI	2.1	0.61	0.37	0.27	0.06	ITO/MA_3_Bi_2_I_9_/Spiro‐OMeTAD/MoO_3_/Ag	245
AgBi_2_I_7_	1.87	0.56	3.30	0.67	1.22	ITO/TiO_2_/mp‐TiO_2_/absorber/P3HT/Ag	265
Cs_2_AgBiBr_6_	2.21	0.98	3.93	0.63	2.43	FTO/c‐TiO_2_/mp‐TiO_2_/absorber/Spiro‐OMeTAD/Au	266
CsBi_3_I_6_	1.77	0.31	3.4	0.38	0.40	FTO/c‐TiO_2_/mp‐TiO_2_/absorber/P3HT/Ag	143
C_6_H_5_NBiI_4_	1.98	0.62	2.71	0.54	0.9	FTO/c‐TiO_2_/mp‐TiO_2_/absorber/ZrO_2_/C	242
(H_3_NC_6_H_12_NH_3_)BiI_5_	2.1	0.40	0.12	0.43	0.03	FTO/c‐TiO_2_/mp‐TiO_2_/absorber/Spiro‐OMeTAD/Au	143
Cs_3_Bi_2_I_9_	2.03	0.02	0.18	0.37	0.02	FTO/TiO_2_/mp‐TiO_2_/absorber/P3HT/Ag	143
Cs_3_Bi_2_I_9_	2.2	0.85	2.15	0.6	1.09	FTO/TiO_2_/mp‐TiO_2_/absorber/Spiro‐OMeTAD/Ag	111

### 7.4 Antimony‐Based Perovskites

Antimony is another potential candidate for replacing lead in perovskite solar cells. Antimony has the ability to form +3 ions with valence electronic configuration similar to that of divalent Pb^2+^. The trivalent Sb^3+^ has a small ionic radius (76 pm) as compared to that of divalent metal cation Pb^2+^ (119 pm) and Sn^2+^ (110 pm), having comparable electronegativity of (2.05) in comparison to Pb (2.33) and Sn (1.96). Antimony‐based lead‐free perovskites form a 0D dimer or a 2D layered structure with the typical basic formula A_3_Sb_2_X_9_ where A is an organic or inorganic cation and X is a halogen.^140^ The choice of cationic or anionic species determines the structure and dimensions of antimony‐based perovskites used as light absorbers. In addition to it, the employed processing technique also effects the dimensions of the synthesized products. Cs_3_Sb_2_I_9_ has an inclination to a 0D dimer form if it is prepared by solution process whereas it prefers a 3D layered structure when prepared through a solid‐state or gas phase reaction. Saparov et al.^140^ carried out thin film preparation and characterization of Cs_3_Sb_2_I_9_ thin films as light absorber in perovskites solar cell. Cs_3_Sb_2_I_9_ film exists in two forms, viz., a 0D dimer form and a 2D layered form. The 0D dimer form of Cs_3_Sb_2_I_9_ is prepared through reactions of CsI and SbI_3_ in stoichiometric ratio of 3:2 in polar solvents. This film has an intense orange color and is stable under ambient air with an indirect bandgap of 2.06 eV whereas 2D layered films of Cs_3_Sb_2_I_9_ are obtained through a solid‐state or gas phase reactions, that is, by sequential deposition of CsI film through evaporation followed by annealing in SbI_3_ vapor. The layered films display red color with a direct bandgap of 2.05 eV, high absorption coefficient of 10^5^ cm^−1^, and ionization energy of 5.6 eV with better stability in air. However, SPCE values of the perovskites solar device with layered forms of Cs_3_Sb_2_I_9_ as light absorber have minimal values of SPCE close to 1% with *V*_OC_ of 0.30 V and a *J*_SC_ below 0.1 mA cm^−2^ indicating a very low overall photovoltaic performance attributed to the presence of deep defects that promote nonradiative recombination. Boopathi et al.^181^ synthesized 0D dimer form of Cs_3_Sb_2_I_9_ as a light absorber and reported a SPCE of 0.84%, *J*_SC_ of 2.91 mA cm^−2^, *V*_OC_ of 0.60 V, and FF of 0.48 for Cs_3_Sb_2_I_9_ with addition of HI.^181^

A 2D layered perovskite was synthesized by using the mixture Cs^+^ and MA^+^ as the A‐site cation via solution process as opposite to reported by Saparov et al. where A‐site cation is substituted by a smaller cation Rb^+^, a 2D layered phase is achieved due to smaller radius of Rb^+^ (1.72 Å) as compared to that of Cs^+^ (1.88 Å) via solution processing through the reaction of RbI and SbI_3_. Using DFT calculations, the comparison of formation energies of 2D layered and 1D dimer forms of A_3_Sb_2_I_9_ (A‐Cs, Rb) reveals that the formation energy difference of 0.25 eV is higher for Rb‐based perovskites than that of cesium‐based counterparts having this difference equal to 0.1 eV thus clearly indicating the increased inclination of Rb_3_Sb_2_I_9_ for layered phase. The layered perovskites Rb_3_Sb_2_I_9_ achieved a SPCE of 0.66% with *V*_OC_ of 0.55 V, *J*_SC_ of 2.11 mA cm^−2^, and FF of 0.57.^267^ In addition, they show thermal stability up to 250 °C and no phase transition is reported in between −40 and 200 °C. The light absorption coefficient of Rb_3_Sb_2_I_9_ films is greater than 1 × 10^5^ cm^−1^ with an indirect bandgap of 2.1 eV. A direct transition at 2.24 eV was calculated for Rb_3_Sb_2_I_9_ as compared to 2.05 eV for the bandgap of cesium. MA_3_Sb_2_I_9_ only forms a 0D dimer structure. The octahedral anionic metal halide [Sb_2_I_9_]^3−^ surround the MA^+^ cations. Hebig et al. first prepared the flat and thin films of MA_3_Sb_2_I_9_ by spin‐coating process followed by toluene treatment. The obtained thin films show a peak absorption coefficient above 10^5^ cm^−1^ and an optical bandgap of 2.14 eV. The fabricated planar perovskite cell achieved SPCE of 0.49%, *V*_OC_ of 0.90 V, *J*_SC_ of 1.0 mA cm^−2^, and FF of 0.55.^147^ Boopathi et al.^181^ synthesized 0D (MA)_3_Sb_2_I_9_ films for use as light absorbers in perovskite solar cells with HI as an additive. The addition of HI into the films resulted in an increase in light absorption in the visible wavelength regions about 400 nm. The XRD spectra studies revealed that the addition of HI leads to a better crystallinity, phase purity, and quality of the film. It reduces the bandgap thereby enhancing the light absorption toward higher wavelength regions. The achieved values of photovoltaic parameters with or without addition of HI are shown in Table 9. The nonsolvent treatment was investigated to enhance the surface morphology of Sn‐based dimer by using HI‐CB to enhance the heterogenous nucleation of Sb‐based perovskite used as light absorber.^268^ The interlayer of HI‐CB acted as a hydrophobic scaffold for the growth of (CH_3_NH_3_)_3_Sb_2_I_9_ crystals. The interlayer decreases the number of voids and enhances the quality of film. The fabricated films achieved a SPCE of 2.77%.^268^The DFT calculations have revealed that the most stable mixed metal organic–inorganic perovskite MA_2_SbI_6_ has a bandgap of 2.0 eV which is further confirmed by using XRD characterization of MA_2_SbI_6_ as a light absorber that has displayed an optical bandgap of 1.93 eV and good stability in air.^269^

A larger A‐site cation was used to synthesize high‐quality films of 2D layered phase in (CH_3_NH_3_)_3_Sb_2_Cl*_x_*I_9−_ *_x_*. The induction of methylammonium chloride into precursor solutions inhibits the formation of the undesirable 0D dimer phase leading to synthesis of high‐quality films of 2D layered phase that is favorable for application in lead‐free perovskite solar cells. These films achieved a SPCE of 2%.^270^ Similarly, Zuo and Ding synthesized a family of perovskite light absorbers (NH_4_)_3_Sb_2_I*_x_*Br_9−_ *_x_* (0 ≤ *x* ≤ 9).^116^ These materials display good solubility in ethanol. The optical light absorption can be adjusted by adjusting the ratio of I and Br content. The absorption onset for films changes from 558 to 453 nm as *x* changes from 9 to 0. The single crystals of (NH_4_)_3_Sb_2_I_9_ showed a hole mobility of 12.3 cm^2^ V^−1^ s^−1^ and electron mobility of 12.3 cm^2^ V^−1^ s^−1^ achieving a *V*_OC_ of 1.03 V and SPCE of 0.51% only.^116^ The use of methylammonium antimony sulfur diiodide (MASbSI_2_) as light absorber for lead‐free perovskite solar cells was first reported by Nie et al.^271^ The MASbSI_2_ is prepared through spin‐coating and thermal annealing of MAI solution on SbSI under mild temperature conditions. The fabricated MASbSI_2_ as light absorber achieved SPCE of 3.08% under the standard illumination condition of 100 mW cm^−2^. They achieved photovoltaic performance in MASbSI_2_ solar cells as of *J*_SC_ (8.12 mA cm^−2^), *V*_OC_ (0.65 V), FF (0.58), and SPCE of 3.08%. Unencapsulated cells stored in dark ambient conditions (humidity ≈60%, temperature 25 °C) retained 90% of their initial efficiency. The use of chalcogenide and halide mixed perovskite materials can be an effective strategy for fabrication of efficient, cheap, and stable solar cells. A mixed metal layered perovskite Cs_4_CuSb_2_Cl_12_ as a light absorber for perovskite solar cells has been reported.^272^ The layered perovskite Cs_4_CuSb_2_Cl_12_ is formed by incorporating Cu^2+^ and Sb^2+^ cations into layers that has a bandgap of 1 eV and conductivity is one order of magnitude greater than MAPbI_3_. Cs_4_CuSb_2_Cl_12_ has high photo and thermal stability and resistance to humidity. The achieved photovoltaic properties promise the excellent use of this material in optical light absorbing layer for perovskite solar cells.^272^

**Figure 15:**
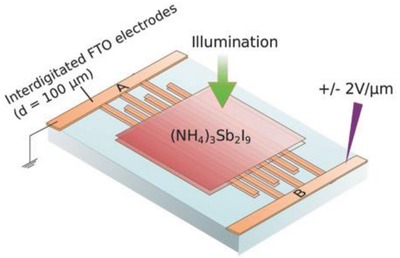
Schematic of the device configuration used for switchable photovoltaic study using (NH_4_)_3_(Sb_(1−_ *_x_*_)_Bi*_x_*)_2_I_9_ perovskite material. Reproduced with permission.^275^ Copyright 2018, Wiley‐VCH.

The normal (n‐i‐p) structured solar cells show better photovoltaic performance as compared to inverted structures. Baranwal et al. have proved it by making a comparison between the normal [n‐i‐p‐Tio_2_‐perovskite‐Spiro‐OMeTAD) and inverted [p‐i‐n‐NiO‐perovskite‐PCBM) structures.^273^ ABX_6_ compounds can form perovskite like 3D crystals frameworks like bromoantimonate (V) (N‐EtPY) (SbBr_6_) with short interhalide contacts.^295^ ASbBr_2_ is a black crystalline solid with an optical bandgap of 1.65 V that is much lower than that of conventional MAPbBr_3_ of 2.3 eV. The planar cells with standard architecture using P3HT as a HTM layer displayed better photovoltaic parameters as *J*_SC_ (5.1 mA cm^−2^), *V*_OC_ (1.285 V), FF (0.58), and SPCE of 3.8% whereas the inverted architecture using a double‐layer PDI as ETL films is fabricated by depositing first by spin‐coating from chlorobenzene solution followed by evaporation of additional layers of the material in vacuum and has shown *J*_SC_ of 5.1 mA cm^−2^, *V*_OC_ of 1.030 V, FF of 0.58, and SPCE of 3.1% only.^274^ The effect of substitution of antimony (Sb) with bismuth (Bi) in a 2D mixed layered perovskite (NH_4_)_3_(Sb_1−_ *_x_*Bi*_x_*)_2_I_9_ as light absorber has been investigated extensively. The partial substitution of Sb with Bi did not change the structure of the crystal but enhanced the volume of the unit cell. The XRD patterns did not show any impurity phase with Bi addition but peaks shift toward lower angles as content of Bi increases showing an increase in unit cell size due to induction of bulkier bismuth cation. The films showed typical features of direct bandgaps due to strong absorption above 2.7 eV and indirect bandgaps because of absence of photoluminescence with long carrier lifetimes. The absorption coefficient increases due to increase in density of states in conduction band whereas bandgap reduces from 2.27 to 2.16 eV^275^ for 5% Bi film due to higher spin–orbit coupling .Bismuth pushes the conduction band downward as predicted by DFT calculations. It also shifts the valence band downward, thereby enhancing the ionization potential values from 5.78 to 5.9 eV for incorporation of 50% bismuth content. The urbach energies also showed a decrease with an increase in bismuth content. The carrier lifetimes do not follow a particular trend with increase in Bi incorporation in the perovskite film as 184 ± 8 ns (0% Bi), 94 ± 25 ns (20%Bi), 149 ± 12 ns (40% Bi), 91 ± 13 ns (50% Bi) as there is decrease in deep defects near the conduction band side due to addition of Bi but simultaneously there is an increase in defects near the valence band. The AC Hall measurements predicted the p‐type conduction band behavior for (NH_4_)_3_Sb_2_I_9_ with a carrier concentration of 3.95 × 10^15^ cm^−3^ and mobility of 0.5 ± 0.5 cm^2^ V^−1^ s^−1^. The carrier density is reduced by incorporating 10 and 20% of Bi owing to increase in mobility that got doubled to more than 1 cm^2^ V^−1^ s^−1^ thus the material undergoes a p‐to‐n transition for higher Bi contents (40%, 50%) that clearly indicates the changing nature of defects in the material. Therefore, the films show both p and n‐type regions. In order to increate p and n regions, electrical poling was used to adjust the load composition of the film by creating ionic drift. The unpoled (NH_4_)_3_Sb_2_I_9_ (p‐type) showed linear photocurrent voltage relationship. The device was negatively poled by applying a bias of −2 V µm^−1^ to electrode B under illumination by a blue LED (455 nm, power 1.4 mW mm^−2^) for 2 min.^275^ The *V*–*I* curves after negative poling indicates photovoltaic effect with *V*_OC_ close to 200 mV which flipped to −0.2 V on ± poling.^275^ The material exhibited measurable photocurrent densities at short‐circuit conditions. The directions of dark and photocurrent densities were opposite resulting in a switch of current direction on illumination due to presence of opposite fields in the same compound. A negative voltage close to −0.6 V is required to achieve zero current condition in dark as opposed to +0.2 V required under illumination. **Table** **10** shows some photovoltaic parameters of antimony‐based lead‐free perovskites. The device configuration is shown in **Figure** **15** for the switchable photovoltaic device containing (NH_4_)_3_(Sb_(1−_ *_x_*_)_Bi*_x_*)_2_I_9_ perovskite material.^275^

**Table 10:** Antimony‐based lead‐free perovskites used as light absorber

Light absorber	*E*_g_	*J*_SC_	*V*_OC_	FF	SPCE	Architecture	Ref.
Cs_3_Sb_2_I_9_	2.05	<0.1	0.31	–	<1.0	FTO/C‐TiO_2_/absorber/PTAA/Au	140
Cs_3_Sb_2_I_9_	2.30	2.34	0.62	0.46	0.67	ITO/PEDOT:PSS/absorber/PC_61_BM/C_60_/BCP/Al	181
Cs_3_Sb_2_I_9_+HI	2.0	2.91	0.60	0.48	0.84	ITO/PEDOT:PSS/absorber/PC_61_BM/C_60_/BCP/Al	181
Rb_3_Sb_2_I_9_	2.1	2.11	0.55	0.57	0.66	FTO/C‐TiO_2_/mp‐TiO_2_/absorber/PolyTPD/Au	267
MA_3_Sb_2_I_9_	2.14	1.0	0.90	0.55	0.49	ITO/PEDOT:PSS/absorber/PC_61_BM/nano‐ZnO/Al	147
MA_3_Sb_2_I_9_	2.20	3.81	0.64	0.45	1.11	ITO/PEDOT:PSS/absorber/PC_61_BM/CO_60_/BCP/Al	181
MA_3_Sb_2_I_9_+HI	1.95	5.41	0.62	0.60	2.04	ITO/PEDOT:PSS/absorber/PC_61_BM/CO_60_/BCP/Al	181
(NH_4_)_3_Sb_2_I*_X_*Br_9−_ *_x_*	2.27	1.15	1.03	0.42	0.51	ITO/PEDOT:PSS/absorber/PC_61_BM/Al	116
MASbSI_2_	2.03	8.12	0.65	0.58	3.08	FTO/BL/mp‐TiO_2_/absorber/PCPD/TBT/PEDOT:PSS/Au	271
Cs_4_CuSb_2_Cl_12_	1.0	–	–	–	0.30	–	272
(N‐EtPY)SbBr_6_ (standard)	1.65	5.1	1.285	0.58	3.8	ITO/C‐TiO*_X_*/absorber/P3HT/Au	274
(N‐EtPY)SbBr_4_ (inverted)	1.65	5.1	1.030	0.58	3.1	ITO/PEDOT:PSS/absorber/PD1/Ag	274
CH_3_Sc(NH_2_)_2_SbA_3_	2.41–3.34	–	–	–	–	–	275

### 7.5 Copper‐Based Perovskites

The divalent Cu^2+^ cation is another suitable element for Pb^2+^ substitution as Cu^2+^ has nontoxic nature. Cu^2+^ has a small ionic radius (73 pm) as compared to Pb^2+^ (119 pm) and Sn^2+^ (110 pm). The divalent Cu^2+^ is more stable in air than Sn^2+^ and Ge^2+^.^135^, ^136^ Cu‐based perovskites usually form 2D layered perovskite structures owing to their smaller ionic radii with general formula (RNH_3_)_2_CuX_4_ where RNH_3_^+^ can be aliphatic or aromatic cation and X is a halogen. They can be easily prepared under suitable conditions by solution method. A 2D cupric perovskite solar cell [p‐F‐C_6_H_5_C_2_H_4_‐NH_3_]_2_CuBr_4_ and (CH_3_(CH_2_)_3_NH_3_)_2_‐CuBr_4_ with absorption range from 300 to 750 nm has been reported. The achieved SPCE values of the fabricated perovskite solar cell are 0.51 and 0.63%, respectively, with good air stability of less than 5% decrease of efficiencies after 1 d in air with humidity of 50% without encapsulation. The reported photovoltaic parameters of the fabricated device are shown in Table 10.^135^ The solar cells based on MA_2_CuCl*_x_*Br_4−_ *_x_* have been investigated in order to study the photovoltaic performance and stability of Cu‐based mixed halides. By tuning Cl/Br ratio, the optical absorption can be extended in the near‐infrared region. The small quantity of Cl^−^ enhances the stability and crystallization of the perovskite material. Among all the investigated light absorbers, the highest SPCE of 0.17% is achieved using MA_2_CuCl_2_Br_2_ as light absorber. The minimal values of SPCE are attributed to reduction of Cu^2+^ and low absorption coefficient. The formation of Cu^2+^ ions was found to be responsible for the green photoluminescence of this material. (MA)_2_CuCl_2_Br_2_ and (MA)_2_CuCl_0.5_Br_3.5_ are found to be more stable under ambient conditions. The achieved values of photovoltaic parameters are shown in Table 10.^136^ It has been found that adding a small amount of CuBr_2_ into MAPbI_3_ remarkably enhances its morphology and efficiency but it is still under investigation whether Cu^2+^ can actually act as substitute for Pb^2+^.^276^

**Figure 16:**
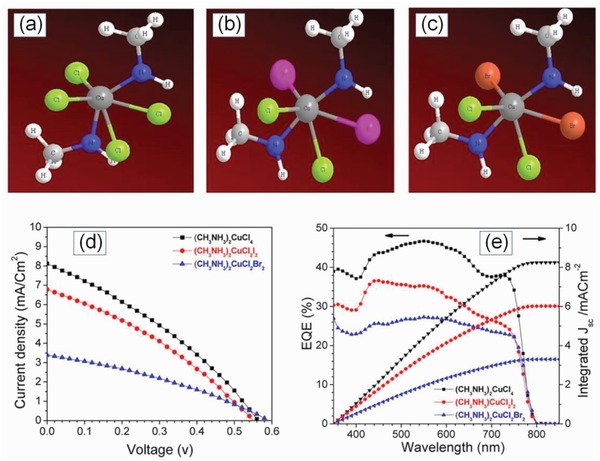
Chemical structures of perovskite solar cells using a) (CH_3_NH_3_)_2_CuCl_4_, b) (CH_3_NH_3_)_2_CuCl_2_I_2_, and c) (CH_3_NH_3_)_2_CuCl_2_Br_2_ powders. d) Current–voltage curve and e) EQE spectra of solar cells. Reproduced with permission.^277^ Copyright 2018, American Chemical Society.

Li et al. investigated and characterized highly stable Cu‐based perovskite films C_6_H_4_NH_2_CuBr_2_I exhibiting extraordinary hydrophobic behavior with a contact angle of ≈90°. The XRD patterns of the perovskite films did not report any change even after 4 h of being immersed in water. The UV absorption of these films revealed their excellent absorption over the entire visible spectrum with low values of SPCE of ≈0.5% attributed to low absorption coefficient and heavy mass of holes.^137^ The other Cu‐based perovskite solar cells (CH_3_NH_3_)_2_CuCl_4_ and (CH_3_NH_3_)_2_CuCl_2_X_2_ [X = I, Br] were fabricated through grinding milling process by Elseman and team and on characterization by XRD reveals that (CH_3_NH_3_)_2_CuCl_2_ has monoclinic crystal structure and (CH_3_NH_3_)_2_CuCl_2_Br_2_ is crystallized with an orthorhombic structure. The tolerance factor and octahedral factor calculated for (CH_3_NH_3_)_2_CuCl_4_ were found to be 1.004 and 0.403, respectively, by assuming the ionic radius of methylammonium to be 18 pm. The calculated values are out of the optimum range of 0.8 < *t* < 0.9 and 0.42 < *u* < 0.895 for a stable 3D perovskite structure thus it crystallizes into 2D structures.^277^ It has been observed that the substitution of Cl^−^ with I^−^ or Br^−^ has different effects on bond angles, unit cell dimensions, and ionic radius. The achieved photovoltaic parameters are depicted in **Table** **11**. The low SPCE values of (CH_3_NH_3_)_2_CuCl_2_Br_2_ are due to reduction of Cu^2+^ caused by the higher trap density. The chemical structures and the performance of Cu‐based perovskite solar cells (a) (CH_3_NH_3_)_2_CuCl_4_, (b) (CH_3_NH_3_)_2_CuCl_2_I_2_, and (c) (CH_3_NH_3_)_2_CuCl_2_Br_2_ powders, (d) current–voltage curve, and (e) EQE spectra of solar cells are shown in **Figure** **16**.^277^

**Table 11:** Photovoltaic parameters of reported Cu‐based perovskites

Light absorber	*J*_SC_ [mA cm^−2^]	*V*_OC_	PCE	FF	*E*_g_	Architecture	Ref.
(CH_3_(CH_2_)_3_NH_3_)_2_CuBr_4_	1.78	0.88	0.63	0.40	1.76	FTO/C‐TiO_2_/mp‐TiO_2_/absorber/Spiro‐OMeTAD/Ag	135
(p‐F‐C_6_H_5_C_2_H_4_‐NH_3_)_2_CuBr_4_	1.46	0.87	0.51	0.40	1.74	FTO/TiO_2_/absorber/Spiro‐OMeTADLiTFSI/Ag	135
MA_2_CuCl_2_Br_2_	0.22	0.26	0.02	0.32	2.12	FTO/C‐TiO_2_/mp‐TiO_2_/absorber/Spiro‐OMeTAD/Au	136
MA_2_CuCl_0.5_Br_3.5_	0.21	0.29	0.002	0.28	1.8	FTO/C‐TiO_2_/mp‐TiO_2_/absorber/Spiro‐OMeTAD/Au	136
C_6_H_4_NH_2_CuBr_2_I	6.20	0.20	0.46	0.46	1.64	FTO/C‐TiO_2_/mp‐TiO_2_/absorber/ZrO_2_/C	137
(CH_3_NH_3_)_2_CuCl_4_	8.12	0.56	2.41	0.52	2.36	Glass/FTO/TiO_2_/absorber/Spiro‐OMeTAD/Au	277
(CH_3_NH_3_)_2_CuCl_2_I_2_	6.78	0.54	1.75	0.47	1.90	Glass/FTO/TiO_2_/absorber/Spiro‐OMeTAD/Au	277
(CH_3_NH_3_)_2_CuCl_2_Br_2_	3.35	0.58	0.99	0.50	1.04	Glass/FTO/TiO_2_/absorber/Spiro‐OMeTAD/Au	277

### 7.6 Other Potential Candidates for Lead‐Free Perovskites

The alkaline earth metals Be^2+^, Mg^2+^, Ca^2+^, Sr^2+^, and Ba^2+^ have been investigated as an alternative to lead in lead‐free perovskite. However, the optical bandgap of Be^2+^ is too high to be used for PV applications. Mg^2+^ despite having a smaller ionic radius (72 pm) can replace Pb^2+^ (119 pm) to form a stable magnesium‐based perovskite.^278^, ^279^ The replacement of Pb^2+^ by Mg^2+^ results in a lead‐free magnesium‐based AMgX_3_ perovskite that exhibits low effective masses, direct optical absorption coefficients, and direct bandgap tunable within the visible region of electromagnetic spectrum depending upon the size of A‐site cation.^278^, ^279^ The bandgaps of magnesium‐based perovskite AMgI_3_ featured an increasing trend with A‐site cations such as FA^+^, MA^+^, and Cs^+^ having values 0.9, 1.5, and 1.7 eV, respectively.^278^ The study of photoluminescence properties of Eu^2+^‐doped CsMI_3_[M‐Mg, Ca, Sr] perovskite has revealed that the Eu^2+^‐doped CsMgI_3_ and CsSrI_3_ displayed a redshift emission with respect to Eu^2+^‐doped CsCaI_3_ perovskite. Eu^2+^‐doped CsMgI_3_ crystallizes in a distorted hexagonal CsNiCl_3_ structure whereas CsCaI_3_ crystallizes in an orthorhombic GdFeO_3_ structure and CsSrI_3_ crystallizes in a filled PuBr_3_ structure.^280^

**Figure 17:**
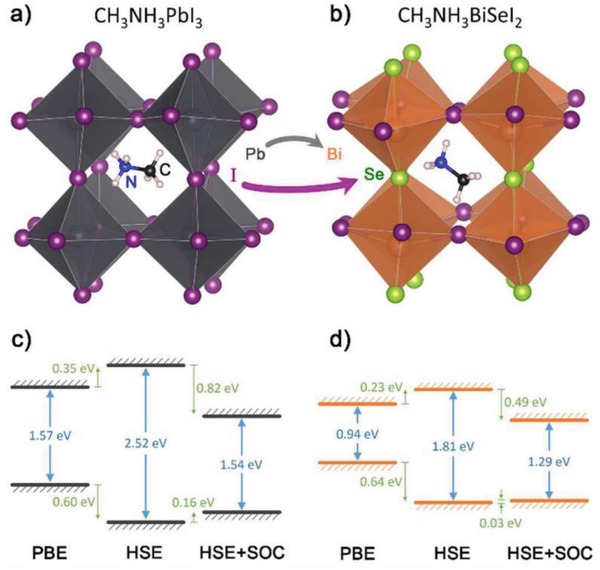
a,b) Atomic structures of CH_3_NH_3_PbI_3_ and CH_3_NH_3_BiSeI_2_ and a schematic illustrating the split‐anion approach to replacing Pb in CH_3_NH_3_PbI_3_. c,d) The calculated bandgaps of CH_3_NH_3_PbI_3_ and CH_3_NH_3_BiSeI_2_, respectively, using improved methods from PBE, HSE to HSE+SOC. The alignment of the band edge positions was obtained by assuming that the reference potentials from different methods are the same. Reproduced with permission.^295^ Copyright 2016, Royal Society of Chemistry.

The divalent Ca^2+^ has an ionic radius (100 pm) comparable to that of Pb^2+^ (119 pm)^281^, ^282^ whereas Sr^2+^ (118 pm) has a similar ionic radii to Pb^2+^ (119 pm).^283^ The divalent Ba^2+^ has an ionic radii of 135 pm larger than that of Pb^2+^ (119 pm).^283^ The DFT calculations have reported bandgaps of MACaI_3_, MASrI_3_, and MABaI_3_ to be 2.95, 3.6, and 3.3 eV, respectively.^281^, ^283^ However, MASrI_3_ and MABaI_3_ do have large bandgaps leading to light absorption in UV region.^281^, ^283^ The replacement of Pb^2+^ by Ca^2+^ and Sr^2+^ in MAPbI_3_ perovskite thin films has reported an increase in long carrier lifetime and fill factors of the devices reaching 0.85.^284^ Transition metals such as Ti, V, Mn, Ni, Pd, Fe, Cu, Zn, Cd, and Hg have been researched extensively for lead replacement in lead‐free perovskite. The crystals of CsNiX_3_ perovskite have been synthesized by hydrothermal method having BaNiO_3_ structure consisting of a face‐sharing NiX_6_ octahedral separated by CsX_12_ cuboctahedra.^285^ The 2D layered perovskite structure of bis(alkyl ammonium)metal(II) tetrahalide (C*_n_*H_2_ *_n_*_−1_NH_3_)_2_MX_4_ and (α,w) polymethylene diammonium metal (II) tetrahalide NH_3_(CH_2_)*_m_*NH_3_MX_4_ with M—Cd, Cu, Fe, Mn, or Pd and X—Br, Cl have been synthesized and a large single perovskite crystal has been obtained.^286^ The divalent Fe^2+^ has a smaller ionic radius (78 pm) as compared to Pb^2+^ (119 pm) that does not allow the formation of 3D perovskite structure. Iron‐based 2D layered perovskite (CH_3_NH_3_)_2_(FeCl_4_) exhibits a canted anti‐ferromagnetism in a magnetic field of strength greater than 2000 Oe and it also exhibits the phase transition from a high symmetry to a low symmetry.^287^ The magnetic susceptibility of (CH_3_NH_3_)_2_FeCl_3_Br perovskite depends upon the strength of applied magnetic field. Also, the size of halide anion has a direct effect on the canted spin.^288^ Just like Sn‐based perovskite, iron‐based perovskite is also unstable due to oxidation of Fe^2+^ to Fe^3+^.^289^ The divalent rare earth Eu^2+^‐based perovskite (C_4_H_9_NH_3_)_2_EuI_4_ has been synthesized through low‐temperature solid‐state reactions.^290^ The effect of doping of rare earth metal ions such as Eu^2+^, Tm^2+^, and Yb^2+^ in CsAX_3_(A‐Ca,Mg,Sr) perovskite has been investigated extensively.^280^, ^291^, ^292^ Another transition metal gold has been investigated for its potential in a perovskite framework. The optical properties of gold‐based 2D organic mixed Au^I^/Au^III^ layered perovskite have been reported with [AuI_2_]^−^/[AuI_4_]^−^ layers supported by I_3_^−^ ions and appropriate organic dications.^293^ The [NH_3_(CH_2_)_7_NH_3_]_2_ [Au^I^ I_2_] [Au^III^ I_4_](I_3_)_2_ and [NH_3_(CH_2_)_8_NH_3_]_2_ (Au^I^ I_2_) (Au^III^ I_4_)(I_3_)_2_ perovskite exhibited a bandgap of 0.95 and 1.14 eV, respectively. The low bandgaps are attributed to the induced electronic interactions between [Au^I^ I_2_]^−^ and [Au^III^ I_4_]^−^ units and I_3_ ions.^293^ Another transition metal tellurium‐based vacancy order perovskite Cs_2_TeI_6_ has been reported that consists of a face‐centered lattice of [TeI_6_]^4−^ units with Cs^2+^ cations occupying the cuboctahedral position. This material do possess an indirect bandgap, electronic dispersion, and is intolerant to formation of defects that is not suitable for PV applications as per current research.^210^ Transition metal titanium‐based perovskite thin films Cs_2_TiBr_6_ have been prepared through low‐temperature‐based method having a bandgap of 1.8 eV that is comparable to eV of lead halide perovskite, balanced carrier diffusion lengths > 100 nm, and highly stable under environmental stresses. The fabricated device exhibited a SPCE of 3.3%. The incorporation of C_60_ interfacial layer between the Cs_2_TiBr_6_ light absorber thin films and TiO_2_ ETM resulted in a *V*_OC_ of 1.02 V in a reverse scan direction and also enhanced other photovoltaic parameters. The thin films are highly stable under ambient conditions.^294^ By the application of split anion approach to MAPbI_3_, the replacement of Pb^2+^ with Bi^3+^ and I^−^ with Se^−^ or S^−^ is done to maintain the charge neutrality thus resulting in lead‐free perovskite CH_3_NH_3_BiSeI_2_, and CH_3_NH_3_BeSI_2_ has been reported exhibiting a direct bandgap of 1.3–1.4 eV suitable for photovoltaic applications.^295^ **Figure** **17** shows the atomic structure and bandgap diagram of CH_3_NH_3_PbI_3_ and MABiSeI_2_ and a schematic illustrating the split‐anion approach to replace Pb in CH_3_NH_3_PbI_3_.^295^

Lead‐free perovskite with mixed chalcogen and halogen anion AB(Ch, X)_3_ where A: Ca or Ba, B: Sb or Bi, X: halogen, Ch: chalcogen has been investigated by using DFT calculations and solid‐state reactions that revealed their thermodynamically unstable nature, that is, their liability to decompose into ternary or binary secondary phases or form phases with nonperovskite structure. The synthesis of mixed perovskite with chalcogen and halogen anions has not been possible due to their unstable nature.^296^ The bandgaps of chalcogenide perovskite CaTiS_3_, BaZrS_3_, CaZrSe_3_, and CaHfSe_3_ with a distorted structure are within the suitable range for photovoltaic performance. Due to their suitable optical absorption properties, chalcogenide perovskite can be the potential candidates to combat the instability and toxicity issues.^297^ The synthesis of polycrystalline chalcogenide perovskite BaZrO_3_, CdZrO_3_, SrTiS_3_, SrZrS_3_, and BsZr(O*_x_*I_3−_ *_x_*)_3_ has been reported by sulfonization of oxide perovskite by CS_2_. The BaZrS_3_ exhibited a distorted perovskite structure as evident from XRD pattern, with an optical absorption from UV to visible region. The perovskite material displayed photoluminescence in visible region and has an excellent stability in ambient air as compared to lead‐based halide perovskites.^298^ The quaternary halide double perovskite employing lanthanides (La^3+^, Ce^3+^, Pr^3+^, Nd^3+^, Sm^3+^, Eu^3+^, Gd^3+^, Dy^3+^, Er^3+^, Tm^3+^, Lu^3+^) and actinides (Pu^3+^, Am^3+^, Bk^3+^) has been investigated but no PV performance has been reported.^299^, ^300^ **Table** **12** summarizes the stability of lead‐free perovskites.

**Table 12:** Stability of lead‐free perovskites

Light absorber	SPCE	Stability	Ref.
MASnI_3_+SnF_2_	2.14%	200 h under 1 sun degradation conditions (AM 1.5, 100 mW cm^−2^)	194
(FA)_0.75_(MA)_0.25_SnI_3_	8.12%	≈80% of SPCE over a period of 400 h, stored in a glove box filled with nitrogen	150
MASnIBr_1.8_Cl_0.2_	3.1%	Average lifetime less than 100 ps	191
FASnI_3_+SnF_2_ pyrazine	4.8%	Stable performances for over 100 d having 98% of its initial efficiency	195
FASnI_3_	6.23%	Stable efficiency of 6% for more than 100 s	172
en[FASnI_3_]	7.14%	Unencapsulated device continues to have 96% of initial SPCE after 1000 h	208
(PEA)_2_(FA)_8_Sn_9_I_28_	5.94%	Unencapsulated devices display performance without significant decay in SPCE over 100 h	156
FASnI_3_+EDAI_2_ (1%)	7.4%	Device stored in glove box displays maximum SPCE of 8.9% for over 1400 h with only slight reduction for storage beyond 2000 h	203
FASnI_3_	5.5%	Encapsulated devices exhibit stability over 1000 h under continuous 1 sun illumination encompassing UV region	167
CsSnIBr_2_+HPA	3%	Exhibits stable efficiency for 77 d and power output within 9 h at high temperature up to 473 K	193
(PEA)_2_GeI_4_	–	2D structure is more stable than 3D MAGeI_3_ in air.	234
BA_2_MA*_n_*_−1_MnI_3_ *_n_*_+1_	1.94–2.53%	2D structure is more stable as compared to 3D counterparts.	235
MA_3_Bi_2_I_9_	0.356%	Exhibits air and moisture stability for more than 60 d	264
MA_3_Bi_2_I_9_ + NMP	0.31%	Unencapsulated device exhibits 88% of SPCE to relative 50–60% humidity for 30 d	244
MA_3_Bi_2_I_9_	0.26%	Exhibits stability for more than ten weeks under ambient conditions	174
Cs_3_Bi_2_I_9_	8%	Unencapsulated device displays initial SPCE for more than 500 h under 1 sun at 65 °C and relative humidity of 60–70%	141
MA_3_Bi_2_I_9_ (with FPDI ETM)	0.06%	Exhibits limited degradation in SPCE after 17 d storage in ambient atmosphere	245
MA_3_Bi_2_I_9_	0.17%	Exhibits 56% of peak SPCE after 300 h exposure to air	262
Cs_2_AgBiBr_6_	2.43%	Unencapsulated device displays excellent stability to working conditions.	266
AgBi_2_I_7_	1.22%	Exhibits excellent stability for at least 10 d under ambient conditions	265
Cs_2_AgBiBr_6_	–	Degrades after light exposure for two weeks	300
KBiI_4_ H_2_O	–	Exhibits considerable stability in air	252
Cs_2_BiAgBr_6_	–	Exhibits stable SPCE in ambient conditions	254
Cs_2_AgBiBr_6_	–	Exhibits degradation after a period of three weeks on exposure to ambient air and light	256
MA_3_Bi_2_I_9_ via (VASP)	3.17%	Unencapsulated devices display stability for 60 d with 0.1%loss in SPCE	247
Cs_3_Sb_2_I_9_	<1.00%	Increased stability under ambient air in comparison to MAPbI_3_ films stored in same condition	140
MA_2_AgSbI_6_	–	Exhibits stability at room temperature in air with 20–60% humidity for 370 d	269
MA_3_Bi_2_I_9_	0.12%	Exhibits no degradation over a month in devices stored in dark in dry air with humidity less than 10%	111
Cs_3_Bi_2_I_9_	1.09%		
AgBi*_x_*I_3_ *_x_*_+1_	0.60%	Unencapsulated devices exhibited SPCE decreasing at a slow pace, 75% of efficiency even after weeks of storage in a N_2_ filled glove box under ambient light	186
1 < *x* < 2.25 (Ag_4_Bi_7_I_25_)			
MA_3_Bi_2_I_9_	0.19%	Displayed excellent stability for more than 400 d upon contact with 50% humidity and air at room temperature	127
MASbSI_2_	3.08%	Unencapsulated devices continues to have 90% of initial SPCE for a period of 15 d when stored in dark with 60% humidity at 25 °C	271
(p‐F‐C_6_H_5_C_2_H_4_‐NH_3_)_2_ CuBr_4_ (CH_3_(CH_2_)_3_NH_3_)_2_CuBr_4_	1.74%	Encapsulated device displays stability in air after 1 d in air with 50%of humidity	135
	1.76%		
C_6_H_4_NH_2_CuBrI	1.64%	Device exhibited hydrophobic behavior with a contact angle of 90° and unchanged XRD patterns after 4 h of water immersion	137
Cs_2_TiBr_6_	3.3%	Unencapsulated films displayed 94% of SPCE after 14 d at 70 °C, 30% RH and ambient light illumination retained 85% of efficiency	294
RbSn_0.5_Ge_0.5_I_3_	–	The activation barrier for water penetration is 0.23 eV in a humid environment that is much higher than for MAPbI_3_ (0.09 eV).	233
CsGeI_3_	0.11%	Stable up to 350 °C	144
MAGeI_3_	0.20%	Stable up to 250 °C	
FAGeI_3_		Stable up to 250 °C	
MASnI_3_	5.8%	Continuous to have 80% of initial SPCE in first 12 h in a properly sealed nitrogen glove box	106
MA_3_Bi_2_I_9_	0.19%	Films display stability over 40 d on conditional exposure to 50% humidity level at room temperature	127
(NH_4_)_3_Sb_2_I_9_	0.51%	Films retained 80% of initial SPCE when stored in a glove box with O_2_ < 10 ppm and H_2_O < 0.1 ppm for 40 d but when in air with 50% humidity, the films lost their PV performance completely	116

## 8 Recent Research on Lead‐Free Perovskites

In order to explore the potential material whose properties can be tailored to be used as a light absorber in a perovskite solar cell, a lot of research is being carried out at present. Research is going on to explore a perovskite material that is lead‐free, nontoxic, have low fabrication cost, easy fabrication technique, higher SPCE, and better stability in air, moisture, and heat. In an attempt to synthesize a low‐cost lead‐free perovskite solar cell, the CH_3_NH_3_SnBr*_x_*Cl_3−_ *_x_* crystals with a trigonal phase have been synthesized via aqueous solution based method by a reaction between HCl and H_3_PO_2_ without taking into account any protection against moisture. The synthesized crystals exhibit various low‐frequency vibrational modes of Sn—Cl and Sn—Br.^261^ Recent studies of lead‐free perovskite have shown that the hot antisolvent treatment of perovskite film increases its coverage and inhibits electrical shunting in photovoltaic device. Also, the average crystallite size increases due to annealing under a low partial pressure of dimethyl sulfoxide vapor. The topographical and electrical qualities of the perovskite film are enhanced facilitating the fabrication of tin‐based perovskite solar cell with a SPCE of over 7%.^301^ The effect of additives on the stability of lead‐free CsSnI_3_ perovskite films has been studied by using first principle based calculations. It has been reported that the additives effectively passivate the surface and enhance the stability of CsSnI_3_ films. The addition of SnBr_2_ as an additive in CsSnI_3_ films resulted in a SPCE of 4.3% with 100 h of stability.^302^

**Figure 18:**
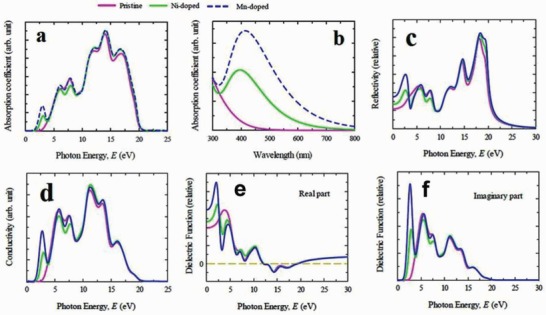
Light absorption spectrum of pristine and metal‐doped (Ni, Mn) CsGeI_3_ perovskite as a function of a) photon energy dependent absorption coefficient, b) wavelength‐dependent absorption coefficient, c) reflectivity, d) conductivity, e) dielectric constant (real part), and f) dielectric constant (imaginary part). Reproduced with permission.^305^ Copyright 2018, Royal Society of Chemistry.

An additional additive formamidinium thiocyanate into quasi‐2D tin perovskite suppresses the oxidation of the material during film formation resulting in a highly crystalline structure with a coarser perovskite grain. The fabricated tin‐based perovskite solar cell reported a SPCE of 8.17% under a reverse scan and a steady‐state efficiency of 7.84%. The fabricated device retained 90% of its efficiency after 1000 h in a glove box filled with nitrogen.^303^ Another study on mixed tin‐germanium perovskite solar cell FA_0.75_MA_0.25_Sn_1−_ *_x_*Ge*_x_*I_3_ has reported that most of the Ge atoms passivate the graded structure of tin perovskite. Upon doping with 5 wt% of Ge, the reported *J*_SC_ (19.8 mA cm^−2^), FF (0.55), and SPCE (4.48%) have shown an increasing trend as compared to 0 wt% of Ge. On further increasing the doping of Ge, the photovoltaic parameters have shown a decreasing trend. The doping of Ge also enhances the stability in air as compared to the nondoped sample.^304^ A recent research on Mn and Ni‐doped CsGeI_3_ perovskite has revealed the effect of doping of Mn and Ni in CsGeI_3_ perovskite that has resulted in enhancement of optical absorption and photoconductivity in visible and UV light region. The optical absorption, dielectric constant, and photoconductivity of Mn‐doped CsGeI_3_ perovskite are larger than that of Ni‐doped counterpart. The Mn‐doped CsGe_1−_ *_x_*Mn*_x_*Cl_3_ perovskite exhibited the potential properties that make it best among all the inorganic pure and metal‐doped CsGeI_3_ perovskite. **Figure** **18** shows the light absorption spectrum of pristine and metal‐doped (Ni, Mn) CsGeI_3_ perovskite as a function of: (a) photon‐energy‐dependent absorption coefficient, (b) wavelength‐dependent absorption coefficient, (c) reflectivity, (d) conductivity, (e) dielectric constant (real part), and (f) dielectric constant (imaginary part).^305^

In a recent study, lead‐free bismuth‐based perovskite CH_3_NH_3_BiX_3_ (X_3_‐I_2_Te, I_2_S, I_2_Se) as a light absorber has been investigated by using first principle calculations. The study has confirmed that CH_3_NH_3_BiX_3_ (X_3_‐I_2_Te, I_2_S, I_2_Se) perovskites are nontoxic in nature exhibiting a high optical absorption in the visible region. These properties pave the way for use of such bismuth‐based perovskite as a light absorber as an alternative to lead‐based CH_3_NH_3_PbI_3_ perovskite in photovoltaic applications.^306^ In another study, lead‐free mixed chalcogen halide perovskite material MABiI_2_S have been synthesized and characterized for its physical and optical properties that revealed a low bandgap of 1.52 eV suitable for optical absorption in the visible spectrum. The fabricated material exhibited an absorption up to over 1000 nm.^307^ The concentration of perovskite solution (0.15–0.30 m) has an effect on the crystallization in MA_3_Bi_2_I_9_ films. Also, the speed of rotation during spin‐coating process determines the layer coverage. The SPCE of the fabricated cells enhances from 0.004 to 0.17% after processing. The fabricated device has exhibited a *V*_OC_ of 0.72 V after 48 h.^308^ Lead‐free inorganic AgBiI_4_ as a light absorber has been prepared by solution method of thin films. The AgBiI_4_ films have been fabricated by 0.6 m solution and annealed at 150 °C. The films exhibited a better morphology with a thermal stability and photostability than that of MAPbI_3_. The fabricated PSC exhibited 2.1% efficiency. The devices displayed long‐term stability and maintained 96% of initial SPCE even after 100 h at relative humidity of 26%.^309^ Lead‐free copper halide perovskite Cs_3_Cu_2_I_5_ have been reported with a 0D structure exhibiting a blue emission (≈445 nm) with a high quantum yield of 90 and 60% for single crystals and thin films. The 0D electronic nature of Cs_3_Cu_2_I_5_ is attributed to a large exciton binding energy of 49 meV and blue emission is demonstrated using solution method Cs_3_Cu_2_I_5_ thin films.^310^ Zinc‐based lead‐free CsZnCl_2_I perovskite 3D thin films have been reported that were deposited at 100 °C by aerosol‐assisted chemical vapor deposition method. The fabricated film displayed absorption peaks at 325 nm excitation covering the entire visible spectrum range.^311^ In another study, perovskite solar cells based on transition metal Ti, Ni, and Cd‐doped BiFeO_3_ as a light absorber with graphene electrode have been investigated. The *V*_OC_ of pure BiFeO_3_, Ti, Ni, Cd‐doped BiFeO_3_ have been reported to be 0.49, 0.77, 0.56, and 0.49 V, respectively. A study of formation of thin films of pure and doped perovskites through three different processes—spinning, dipping, and spray process—has been carried out that revealed that Ti‐based BiFeO_3_ in spinning process have given the best results.^312^ Lead‐free Ti‐based perovskites have been investigated for their photovoltaic behavior.^313^ Transition metal palladium‐based lead‐free perovskite Cs_2_PdBr_6_ nanocrystals have been reported with an average particle diameter of 2.8 nm and a thickness of 1–2 units cells exhibiting a narrow bandgap of 1.69 eV and outstanding stability toward light humidity and heat. The fast anion exchange method has been employed to synthesize Cs_2_PdI_6_ nanocrystals.^314^

Lead‐free (1−*x*) (K_0.44_Na_0.52_Li_0.04_)(Nb_0.91_Ti_0.05_Sb_0.04_)O_3_‐xSmAlO_3_ [*x* = 0, 0.001, 0.004, 0.004, 0.008] ceramics have been synthesized by a solid‐state sintering method. The effect of doping of SmAlO_3_ on the phase structure and electrical properties of all the perovskite composition for reported values of *x* have been investigated thoroughly. From the study of XRD analysis, all the investigated composition reported a perovskite structure at the suitable sintering temperature. The enhanced electrical properties were obtained at the sintering temperature of 1180 °C.^315^ Lead‐free multiferroic (1−*x*) KNbO_3_‐(*x*)CoFe_2_O_4_ composites have been synthesized by employing solid‐state reaction method with *x* (0, 0.25, 0.5, 0.75, 1.0) mol. The careful study of XRD reveals that KNbO_3_ perovskite belong to an orthorhombic system, spinal CoFe_2_O_4_ belong to cubic system, and other compositions of *x* belong to mixed phase of KNbO_3_ and CoFe_2_O_4_. The high‐resolution SEM analysis has shown that the morphology of KNbO_3_ and CoFe_2_O_4_ was modified by CoFe_2_O_4_ content. The composite 0.5KNbO_3_ 0.5 CoFe_2_O_4_ displayed a high value of coercivity and 0.5KNbO_3_ 0.5CoFe_2_O_4_ and 0.75KNbO_3_ 0.75 CoFe_2_O_4_ displayed an enhanced value of dielectric constant.^316^ At present, a lot of research is going on lead‐free double perovskite materials to explore their potential as a light absorber in perovskite solar device. Double perovskite A_2_B′B″X_6_[A‐Cs, MA, B′‐Bi, Sb, B″‐Cu, Ag, X‐Cl, Br, I] have been investigated for their structural, optical, and stability properties.^317^ The vapor‐assisted method has been employed to synthesize double perovskite Cs_2_AgBiB_6_ thin films with better morphology. The better quality of Cs_2_AgBiB_6_ films has a photoluminescence lifetime of 117 ns. The fabricated n‐i‐p perovskite solar cell has reported a SPCE of 1.37% with a better stability of 90% after 240 h of storage under ambient conditions.^318^ The diffusion of X halide anion in lead‐free double perovskite Cs_2_AgBiX_6_ [X‐Cl, Br], Cs_2_AgSbCl_6_, Cs_2_AgInCl_6_ has been investigated by using first principle calculations. The calculated values of formation energy of X‐site vacancies are related to electronic configuration of B‐site cations. The double perovskite Cs_2_AgInCl_6_ is having lowest vacancy formation energy due to unfilled s‐orbital of In^3+^. The hysteresis loss in Cs_2_AgBiBr_6_ solar cells is attributed to the lowest energy barrier for X‐site migration.^319^ Double perovskite lead‐free layered Cs_4_CuSb_2_Cl_12_ have been reported with a bandgap of 1 eV prepared by grinding of precursor salts at ambient conditions. A long range magnetic ordering is displayed by the synthesized perovskite at room temperature that plays a pivotal role in controlling the electronic properties of double perovskite Cs_4_CuSb_2_Cl_16_.^320^ By using first principle calculations, lead‐free double perovskite Cs_2_NaBX_6_[B‐Sb, Bi, X‐Cl, Br, I] have been synthesized and characterized for their electronic and optical properties. The simple solution method has been employed to prepare a layered MA_3_Bi_2_I_9_ perovskite and a composite layer of bismuth tri iodide (BiI_3_). By employing SEM and XRD techniques, the morphology of the active layer has been investigated that has a direct influence on performance of the perovskite device.^321^ The high‐temperature solid‐state reaction method has been employed to prepare polycrystalline material of double perovskite Dy_2_NiMnO_6_ with a monoclinic structure. The high‐temperature condition of the material is attributed to the presence of oxygen vacancies making it viable to use at different temperatures.^322^

## 9 Conclusion

The research in tin‐based perovskites MASnX_3_ has revealed a direct bandgap of 1.20–1.35 eV, electron mobility of 2320 cm^2^ V^−1^ s^−1^, hole carrier mobility of 322 cm^2^ V^−1^ s^−1^, and long charge carrier diffusion length of more than 500 nm. The alteration of Br^−^/I^−^ ratio in MASnI_3−_ *_x_* Br*_x_* has resulted in large value of *V*_OC_ (0.88 V) in MASnBr_3_ and *J*_SC_ (12.33 mA cm^−2^) in MASnIBr_2_. The absorption band can be tuned by altering the composition of halide anions in MASnX_3_ perovskites. However, Sn‐based perovskites suffer from degradation in air due to oxidation of Sn^2+^ into Sn^4+^. The incorporation of additives has resulted in reduced oxidation and better stability in air. The A‐site cation has a significant effect on photovoltaic performance. The use of diethylammonium (en) and FA^+^ at the A‐site of ASnX_3_ has resulted in wider bandgaps and improved stability. An efficiency of 8% has been achieved for (FA)_0.75_(MA)_0.25_SnI_3_ with a *V*_OC_ (0.61 V) and bandgap (1.33 eV). Germanium‐based perovskites do have an optical bandgap of 1.63 eV, excellent hole and electron conducting behavior, and better stability in air. Using DFT calculations, it has been reported that with increase of size of halide anion, the bandgaps have decreasing values of 3.7, 2.81, and 1.61 eV, respectively. The replacement of the iodide content in AGeI_3_ by bromide results in enhanced photovoltaic performance and stability to a slight extent. Mixed Ge‐based perovskite RbSn_0.5_Ge_0.5_I_3_ exhibits a better optical absorption and effective masses for higher carrier mobility and good stability in water. By engineering the size of A‐site cation, its doping with another suitable cation and size of halide anion, it is possible to fabricate a Ge‐based perovskite as an efficient light absorber. Although, bismuth‐based perovskite (MA)_3_Bi_2_I_9_ has displayed low values of solar power conversion efficiencies up to 1.64 eV up to now, yet they have exhibited excellent stability in ambient air at room temperature and against exposure to humidity .The morphology of (MA)_3_Bi_2_I_9_ films can be enhanced by addition of various concentration of NMP into the precursor solution that not only controlled the rate of crystallization but also enhanced the efficiency and stability in a relative humidity of 50–60%. The concentration of perovskite solution and substrate temperature also impacts its efficiency and stability. The wide bandgaps of lead‐free perovskites can be engineered to a narrow bandgap by incorporating triiodide into P(4‐methyl piperidinium)_3_Bi_2_I_9_(MP‐Bi_2_I_9_) that exhibited a bandgap of 1.58 eV in comparison to 1.5 eV of MAPbI_3_. The various deposition methods have a direct influence on morphology of films. Cs_3_Bi_2_I_9_ has displayed an efficiency of 8% with a pure crystalline phase and stability. Bismuth‐based double perovskite like Cs_2_AgBiBr_6_ exhibited an indirect bandgap of 2.19 eV. The DFT calculations have further revealed that the family of 3D double perovskites have optical bandgap in the visible range and low carrier effective masses. Bismuth‐based perovskites can be thoroughly investigated for enhancement in their efficiency as these materials have excellent stability in ambient air and in relative humidity. In antimony‐based perovskites, the size of cationic or anionic species and the employed processing technique determine the structure. When A‐site cation Cs^+^ is replaced by a smaller cation Rb^+^, a 2D layered phase is achieved with a formation energy difference of 0.25 eV in comparison to Cs‐based counterparts. The addition of additive in 0D (MA)_3_Sb_2_I_9_ films has resulted in enhanced light absorption in the visible wavelength regions up to 400 nm. The use of chalcogenide and mixed perovskite materials can be an effective strategy for formation of efficient, cheap, and stable solar cells. Cs_4_CuSb_2_Cl_12_, besides having photo and thermal stability and resistance to humidity, have exhibited excellent photovoltaic properties. There has been a significant effect on photovoltaic parameters on substitution of Sb with Bi in 2D mixed layered perovskites (NH_4_)_3_(Sb_1−_ *_x_*Bi*_x_*)_2_I_9_. By proper substitution of Bi into antimony‐based perovskites, it is possible to fabricate light harvesters with high efficiency and stability. Copper‐based perovskites usually form 2D layered structure owing to their smaller ionic radii. By proper tuning of Cl^−^/Br^−^ ratio, the optical absorption of Cu‐based perovskites can be extended in the near‐infrared region. The (MA)_2_CuCl_2_Br_2_ and (MA)_2_CuCl_0.5_Br_3.5_ have reported better stability under ambient conditions. The divalent cations of alkaline earth metals like Mg^2+^, Ca^2+^, and Sr^2+^ can be effective replacement for lead. The perovskite solar cells based on transition metals Ti, Ni, and Cd‐doped BiFeO_3_ as a light absorber have displayed *V*_OC_ values of 0.77, 0.56, and 0.49 V, respectively. By suitable selection of A and B‐site cations and halide anions, their alteration in composition and synthesis method, it is possible to fabricate lead‐free perovskites with maximum efficiency and stability without any toxic influence on environment.

## Conflict of Interest

The authors declare no conflict of interest.
